# Prevalence and demographic variation of cardiovascular, renal, metabolic, and mental health conditions in 12 million english primary care records

**DOI:** 10.1186/s12911-023-02296-z

**Published:** 2023-10-16

**Authors:** Jennifer Cooper, Krishnarajah Nirantharakumar, Francesca Crowe, Amaya Azcoaga-Lorenzo, Colin McCowan, Thomas Jackson, Aditya Acharya, Krishna Gokhale, Niluka Gunathilaka, Tom Marshall, Shamil Haroon

**Affiliations:** 1 Institute of Applied Health Research, Health Data Science and Public Health, University of Birmingham, Birmingham, UK; 2 School of Medicine, University of St Andrews, Fife, UK; 3 Clinician Scientist in Geriatric Medicine, Institute of Inflammation and Ageing, University of Birmingham, Birmingham, UK

**Keywords:** Electronic health records, Prevalence, Cardiovascular, Renal, Metabolic, Mental health

## Abstract

**Background:**

Primary care electronic health records (EHR) are widely used to study long-term conditions in epidemiological and health services research. Therefore, it is important to understand how well the recorded prevalence of these conditions in EHRs, compares to other reliable sources overall, and varies by socio-demographic characteristics. We aimed to describe the prevalence and socio-demographic variation of cardiovascular, renal, and metabolic (CRM) and mental health (MH) conditions in a large, nationally representative, English primary care database and compare with prevalence estimates from other population-based studies.

**Methods:**

This was a cross-sectional study using the Clinical Practice Research Datalink (CPRD) Aurum primary care database. We calculated prevalence of 18 conditions and used logistic regression to assess how this varied by age, sex, ethnicity, and socio-economic status. We searched the literature for population prevalence estimates from other sources for comparison with the prevalences in CPRD Aurum.

**Results:**

Depression (16.0%, 95%CI 16.0–16.0%) and hypertension (15.3%, 95%CI 15.2–15.3%) were the most prevalent conditions among 12.4 million patients. Prevalence of most conditions increased with socio-economic deprivation and age. CRM conditions, schizophrenia and substance misuse were higher in men, whilst anxiety, depression, bipolar and eating disorders were more common in women. Cardiovascular risk factors (hypertension and diabetes) were more prevalent in black and Asian patients compared with white, but the trends in prevalence of cardiovascular diseases by ethnicity were more variable. The recorded prevalences of mental health conditions were typically twice as high in white patients compared with other ethnic groups. However, PTSD and schizophrenia were more prevalent in black patients. The prevalence of most conditions was similar or higher in the primary care database than diagnosed disease prevalence reported in national health surveys. However, screening studies typically reported higher prevalence estimates than primary care data, especially for PTSD, bipolar disorder and eating disorders.

**Conclusions:**

The prevalence of many clinically diagnosed conditions in primary care records closely matched that of other sources. However, we found important variations by sex and ethnicity, which may reflect true variation in prevalence or systematic differences in clinical presentation and practice. Primary care data may underrepresent the prevalence of undiagnosed conditions, particularly in mental health.

**Supplementary Information:**

The online version contains supplementary material available at 10.1186/s12911-023-02296-z.

## Background

Cardiovascular, renal, and metabolic (CRM) and mental health (MH) conditions (listed in Box 1) are amongst the most common causes of death and disability globally, [1–5] with MH conditions alone accounting for almost a third of the global burden of years lived with disability [1]. Primary care electronic health records (EHR) databases are routinely used in observational studies of the epidemiology of these long-term health conditions [6]. Clinical Practice Research Datalink (CPRD) Aurum is a relatively new primary care EHR database, with a number of strengths stemming from the richness of the nationally representative routinely collected data, which captures patient demographics, diagnoses, test results, and prescriptions for over 19 million patients [7]. However, there are recognised limitations to EHR data and there are inevitably disparities between self-reported health status and conditions reported in EHRs with variation in case-detection rate according to age, sex and other demographic characteristics. [8–10]. A recent US study found varying agreement between self-reported survey answers and EHR diagnoses data, with 81% positive agreement for type 2 diabetes and 59% positive agreement for depression [9].

Objective clinical investigations are typically used to diagnose CRM conditions (e.g. glycosylated haemoglobin (HbA1c) for diabetes or computed tomography (CT) for strokes), although there is still considerable potential for both under and over diagnosis of these conditions [11]. On the other hand, MH diagnoses are based on clusters of symptoms with an element of subjectivity on the part of the diagnosing clinician, especially in milder cases, and there are also a number of recognised barriers to seeking help for MH conditions including societal stigma and difficulties in asking for and accessing support, which may lead to underdiagnosis [12]. The extent of these barriers is likely to vary according to ethnicity, sex and socio-economic status [13]. Furthermore, conditions that are primarily diagnosed in secondary care, might not be as well captured in primary care records where there is inefficient information transfer between hospitals and GP practices. Studies comparing primary care EHR to hospital episode statistics have shown that only around 60% of hospital admissions for stroke were recorded in primary care EHRs [14]. These factors may lead to disparities between the prevalence of diagnoses in EHRs and screen-detected prevalence estimates for MH conditions across both socio-demographic characteristics and when compared with CRM conditions. However, there is a paucity of research that has examined the extent of these disparities in primary care records for this range of conditions, particularly in CPRD and other UK EHR databases.

It is also valuable to compare the prevalence of health conditions in CPRD Aurum with those from other sources (e.g., national health surveys, screening studies) to understand the strengths and limitations of current and future epidemiological research using CPRD Aurum and other similar EHR databases. Therefore, the primary objective of this study was to describe the prevalence of selected CRM and MH conditions within this database and assess variation in the prevalence of reported conditions by categories of age, sex, ethnicity, and socio-economic deprivation. Secondly, we aimed to compare the prevalence of the conditions in this database against the prevalence within the UK (or similar countries) general population in three other sources in the literature: (1) other primary care EHR databases; (2) self-reports of doctor-diagnosed conditions in nationally representative surveys; and (3) screening studies.

## Methods

### Study design, data source and population

This was a cross-sectional analysis of the CPRD Aurum database, which contains routinely-collected primary care EHRs from 1,444 general practices across England using EMIS Web® patient records software [7]. Clinical observations, diagnoses and treatments are recorded as Read Version 2, SNOMED-CT, and EMIS Web® clinical codes. The full data resource profile has been described elsewhere [7]. A cross-sectional dataset was extracted for analyses using the Data Extraction for Epidemiological Research (DExtER) tool [15]. Data for these analyses included all patients who were alive and permanently registered with a participating practice on 1st January 2020 (this date was chosen so that results would not be influenced by the impact of the SARS-CoV-2 pandemic on primary care activity and data recording). Patients were only included if there were at least 12 months of acceptable data recording prior to the index date (1st January 2020). Acceptable data was determined using the “acceptable patient flag” data quality measure provided by CPRD: (consistent recording of events including date of birth, practice registration date and transfer out date, and valid age and gender) [7, 16]. The dataset includes patients’ year of birth, sex, ethnicity, and their socio-economic status (index of multiple deprivation (IMD) quintile). Results of this cross-sectional analysis were compared against population prevalence of these same conditions determined from a literature review.

### Selection of cardio-renal-metabolic and mental health conditions

A recent Delphi study has identified key conditions that are important to patient and research stakeholders for inclusion in research into patients with multiple long term conditions [17]. From the results of this study, and after discussions within our clinical team and patient advisory group, eight MH and ten CRM conditions were selected for inclusion in our analyses (see Box 1). We included all recommended cardiovascular conditions from the Delphi study except for venous thromboembolic disease as we have focused on chronic rather than acute conditions. We included all recommended “mental health” conditions except for autism and dementia as these are neurodevelopmental and neurodegenerative conditions, respectively. We also added diabetes and chronic kidney disease (CKD) as these are highly prevalent chronic conditions which are closely related to cardiovascular disease.

**Box 1:** Included conditions

Cardio-renal-metabolic (CRM) conditions	Mental health (MH) conditions
Stroke	Depression
Ischaemic heart disease (IHD)	Anxiety
Heart failure	Bipolar disorder
Diabetes (T1DM and T2DM)	Substance misuse
Hypertension	Alcohol misuse
Chronic kidney disease (CKD)	Eating disorders
Heart valve disorders	Post-traumatic stress disorder (PTSD)
Atrial fibrillation (AF)	Schizophrenia
Aortic aneurysms	
Peripheral vascular disease (PVD)	

### Outcome measures in CPRD Aurum

Prevalent cases for all conditions were identified using disease-specific clinical codelists. Codelists were developed through collaboration by a team of clinicians in the Universities of Birmingham and Cambridge using a rigorous, systematic process via the DExtER codebuilder tool, with search strategies recorded using a consistent coding checklist. We began by reviewing all existing Quality and Outcomes Framework (QOF) codelists, [18] and published codelists for UK primary care EHR analyses, including HDRUK Phenotype library, [19] OpenCodelists, [20] and CPRD @ Cambridge Codelists [21]. Lists were adapted or, where they did not exist, created anew for CPRD Aurum using the hierarchical Read code system, the NHS Digital SNOMED CT term browser, [22] and the DExtER codebuilder tool to search for relevant text words for symptoms, diagnoses, clinical findings, and interventions that indicated a diagnosis of condition. Finally, codelists, conventions and queries were reviewed and agreed among the team at regular clinical coding meetings. Codelists can be found at https://github.com/THINKINGGroup/phenotypes.

For hypertension and CKD, prescriptions and clinical biomarkers were also used as a secondary method of determining prevalence estimates. Hypertension was defined (according to the same methods as the Health Survey for England [23] to enable comparison) as prescription of an antihypertensive medication in the six months prior to 1st January 2020, or most recent blood pressure within the past three years > 140/90mmHg. CKD was defined as the most recent estimated glomerular filtration rate (eGFR) < 60ml/min/1.73 m² within the past three years prior to 1st January 2020.

### Outcome measures in comparator sources

A literature review was undertaken to identify, for each condition, three estimates for UK population prevalence:

#### UK primary care electronic records prevalence

- Previous analyses of UK primary care electronic records databases using clinical codes to detect prevalent cases. Where available, QOF data were the ideal comparator as the Quality and Outcomes Framework programme uses data collected from 96% of general practices in England [18]. Practices are financially incentivised via QOF to keep accurate disease registers of patients with specific conditions according to nationally agreed standards. For conditions not included in QOF we used cross-sectional, or cohort studies analysing data from other UK EHRs.

#### Self-reported doctor-diagnosed prevalence

- Prevalence estimates identified from studies using methods other than primary care EHRs for detection of cases that have been diagnosed by a healthcare professional. These estimates primarily came from two large cross-sectional studies: the Health Survey for England (HSE) and the Adult Psychiatric Morbidity Survey (APMS) where a nationally representative sample of the UK population were surveyed face-to-face and asked about their health conditions [14, 23].

#### Screen-detected prevalence

- Prevalence estimates identified from studies that involved screening of a representative sample of the population using a reference standard diagnostic technique. For example, the Health Survey for England (HSE) used HbA1c blood tests from the representative sample to estimate population prevalence of diabetes [23].

### Search strategy

A pragmatic approach was used to identify relevant sources for each condition; where available, prevalence statistics reported within Public Health England fingertips resources, [24] NHS Digital resources, [25] and NICE Clinical Knowledge Summaries were used [26]. Further details of the search strategy can found in Additional file 1. Where QOF, HSE, or AMPS data were not available, PubMed, and Google scholar databases were systematically searched for cross-sectional and longitudinal studies using a Boolean search strategy; “condition name” AND “prevalence” OR “epidemiology”. For EHR prevalence, we added: AND abbreviated and unabbreviated names of these established UK-based primary care EHR databases (e.g., “THIN” and “The Health Improvement Network”). For screen-detected prevalence we added: AND “screening”.

### Study selection criteria

Studies were included if they reported the most recent available prevalence of any of the conditions using cross-sectional or cohort study data (or a meta-analysis of these), representative of the general population prior to 1st January 2020. They were excluded if they contained fewer than 500 patients or were based on a subpopulation within a specific disease. The most recent study within a large and comparable population was selected. This was ideally a UK population study, but if this was not available then studies within European or other high-income countries were used. Further details and methods of data collection for all comparator studies were summarised in Additional file 1, and in Additional Table 1, Additional Table 2, and Additional Table 3 within that additional file.

### Statistical methods

#### CPRD aurum prevalence analysis

Frequencies, percentages, and cross-tabulations were used to describe the prevalence of each condition across the entire population and by sociodemographic characteristics with age, sex, ethnicity groups, and deprivation quintiles all treated as categorical variables. Age at entry was categorised into the following age groups: 0–16, 17–30, 31–40, 41–50, 51–60, 61–70, and ≥ 70 years. Ethnicity was categorised into five groups based on those used in the UK Census: white, Asian, black, mixed, and other ethnicity (which includes Chinese, Middle Eastern and Pacific). Socioeconomic categories were based on the English Index of Multiple Deprivation (IMD) quintiles for the geographic area where the patient lives. Patients with missing data on ethnicity were assigned to a separate “missing” category and included in the regression analysis.

For each point estimate of prevalence, 95% confidence intervals (CI) for proportion were calculated using the Clopper-Pearson exact method [27]. Logistic regression was used to calculate the odds ratios of each condition by sociodemographic characteristics (with mutual adjustment). All statistical analyses were performed using Stata statistical software, V.16 (StataCorp, College Station, Texas, USA). Stata codes used for the analysis are publicly available here: https://github.com/CPRDAurumPrevalenceAnalysis/.

#### Comparator data prevalence analysis

Numerators (number of cases) and denominators (number of people sampled) and details of the data collection methods were extracted from each source identified in the literature review. Population prevalence and 95% confidence intervals for proportions were calculated for each condition in the same way as for the CPRD Aurum analysis.

#### Comparisons between prevalence estimates

For each comparison with the prevalence reported in the literature, a sample was created within CPRD Aurum containing all patients who matched the age profile of that population. For aortic aneurysms the only available comparator was from a screening programme that reported incidence within men in their 65th year, therefore a comparison was made with prevalence of aortic aneurysm in men aged 66 (to allow time for the diagnosis to be recorded in their records). For anxiety, the most appropriate comparator population prevalence estimates only measured prevalence of generalised anxiety disorder. Therefore, a new codelist for generalised anxiety disorder was created within CPRD Aurum for comparison. The prevalence estimates were compared using scatter graphs of observed vs. comparator prevalence using Microsoft Excel.

## Results

### Cross-sectional analysis of primary care EHR

Almost 12.4 million patients within the CPRD Aurum database were eligible for inclusion in this analysis. The median length of follow up in this study was 10.2 years (IQR 4.4–20.9) Males and females were equally represented; 18% of the patients were under 16, 69% were between 16 and 70, and 13% were over 70 years old. Ethnicity was recorded for 80% of patients in the database, and of these 81% were White, 10% were Asian, 5% were Black, 2% were of other ethnicities, and 2% were of mixed ethnicity. Deprivation quintiles were equally distributed (~ 20%). Hypertension, affecting 15% of the study population and depression, affecting 16%, were the most common CRM and MH conditions respectively. The prevalence of those with each CRM and MH condition in the general population and by socio-demographic characteristics are shown in Tables 1 and 2.

**Table 1:** Prevalence of cardio-renal-metabolic conditions overall and by socio-demographic in the CPRD Aurum database, 2020

	All	Aortic aneurysm	Atrial fibrillation (AF)	(CKD) (clinical codes)	CKD (latest eGFR < 60)	Diabetes (Type 1)	Diabetes (all)	Heart failure	Heart valve disorders	Hypertension (clinical codes)	Hypertension (BP > 140/ 90) *	Ischaemic heart disease (IHD)	Peripheral vascular disease (PVD)	Stroke
Number (%)	12,361,554	33,292 (0.27)	260,966 (2.11)	431,791 (3.49)	505,860 (4.09)	60,342 (0.49)	721,301 (5.84)	166,273 (1.35)	169,116 (1.37)	1,885,400 (15.25)	4,003,524 (32.39)	481,846 (3.90)	82,890 (0.67)	207,911 (1.68)
Age (median, IQR)	**39.5 (22.5–58.5)**	76.5 (69.5–83.5)	77.5 (69.5–84.5)	78.5 (70.5–85.5)	79.5 (71.5–85.5)	49.5 (34.5–62.5)	66.5 (55.5–75.5)	76.5 (66.5–84.5)	74.5 (60.5–82.5)	69.5 (58.5–77.5)	59.5 (46.5–71.5)	73.5 (63.5–81.5)	74.5 (66.5–81.5)	72.5 (60.5–81.5)
**Age Categories (years)**														
0–16	**2,215,109 (17.9)**	48 (< 0.01)	24 (< 0.01)	233 (0.01)	88 (< 0.01)	4,026 (0.18)	4,118 (0.19)	726 (0.03)	6,455 (0.29)	2,383 (0.11)	14,987 (0.68)	2,215,109 (0.01)	94 (< 0.01)	1,135 (0.05)
17–30	**2,157,953 (17.5)**	103 (< 0.01)	578 (0.03)	833 (0.04)	842 (0.04)	10,276 (0.48)	13,394 (0.62)	1,542 (0.07)	7,693 (0.36)	18,104 (0.84)	276,207 (12.80)	2,157,953 (0.15)	220 (0.01)	3,093 (0.14)
31–40	**1,808,259 (14.6)**	178 (0.01)	2,018 (0.11)	2,649 (0.15)	2,390 (0.13)	8,774 (0.49)	26,084 (1.44)	2,311 (0.13)	6,993 (0.39)	45,803 (2.53)	374,226 (20.70)	1,808,259 (0.31)	490 (0.03)	5,744 (0.32)
41–50	**1,676,254 (13.6)**	377 (0.02)	5,607 (0.33)	8,149 (0.49)	7,082 (0.42)	9,426 (0.56)	67,810 (4.05)	5,266 (0.31)	9,142 (0.55)	137,914 (8.23)	574,506 (34.27)	1,676,254 (1.03)	1,784 (0.11)	12,141 (0.72)
51–60	**1,704,779 (13.8)**	1,128 (0.07)	18,211 (1.07)	27,555 (1.62)	25,646 (1.50)	11,039 (0.65)	142,704 (8.37)	15,240 (0.89)	16,311 (0.96)	336,486 (19.74)	842,262 (49.41)	1,704,779 (3.45)	8,173 (0.48)	27,771 (1.63)
61–70	**1,254,678 (10.2)**	7,234 (0.58)	40,984 (3.27)	62,122 (4.95)	66,026 (5.26)	8,157 (0.65)	175,564 (13.99)	27,881 (2.22)	24,365 (1.94)	447,809 (35.69)	768,883 (61.28)	1,254,678 (8.43)	18,915 (1.51)	40,294 (3.21)
70+	**1,544,522 (12.5)**	24,224 (1.57)	193,544 (12.53)	330,250 (21.38)	403,786 (26.14)	8,644 (0.56)	291,627 (18.88)	113,307 (7.34)	98,157 (6.36)	896,901 (58.07)	1,152,453 (74.62)	1,544,522 (18.84)	53,214 (3.45)	117,733 (7.62)
**Sex**														
Male	**6,224,433 (50.4)**	27,081 (0.4)	150,874 (2.4)	180,645 (2.9)	222,605 (3.6)	34,163 (0.5)	401,806 (6.5)	94,485 (1.5)	81,494 (1.3)	931,858 (15.0)	1,922,607 (30.9)	298,464 (4.8)	52,627 (0.8)	108,374 (1.7)
Female	**6,137,121 (49.6)**	6,211 (0.1)	110,092 (1.8)	251,146 (4.0)	283,255 (4.6)	26,179 (0.4)	319,495 (5.1)	71,788 (1.2)	87,622 (1.4)	953,542 (15.3)	2,080,917 (33.4)	183,382 (2.9)	30,263 (0.5)	99,537 (1.6)
**Ethnicity**														
White	**7,919,608 (64.1)**	27,395 (0.4)	213,108 (2.7)	337,649 (4.3)	398,122 (5.0)	45,195 (0.6)	493,914 (6.2)	129,746 (1.6)	130,422 (1.6)	1,417,789 (17.9)	2,942,191 (37.2)	375,726 (4.7)	67,323 (0.9)	159,183 (2.0)
Asian	**1,024,958 (8.3)**	658 (0.1)	5,149 (0.5)	18,971 (1.9)	22,271 (2.2)	3,226 (0.3)	96,601 (9.4)	8,210 (0.8)	7,310 (0.7)	119,782 (11.7)	267,799 (26.1)	32,367 (3.2)	2,507 (0.2)	9,435 (0.9)
Black	**508,581 (4.1)**	385 (0.1)	2,945 (0.6)	14,181 (2.8)	11,096 (2.2)	2,373 (0.5)	43,021 (8.5)	4,882 (1.0)	3,982 (0.8)	87,138 (17.1)	158,031 (31.1)	9,068 (1.8)	1,612 (0.3)	6,396 (1.3)
Mixed ethnicity	**207,281 (1.7)**	114 (0.1)	860 (0.4)	2,709 (1.3)	3,597 (1.7)	707 (0.3)	8,524 (4.1)	981 (0.5)	1,139 (0.5)	16,856 (8.1)	41,572 (20.1)	2,738 (1.3)	375 (0.2)	1,430 (0.7)
Other ethnicity	**171,985 (1.4)**	112 (0.1)	838 (0.5)	1,488 (0.9)	1,879 (1.1)	447 (0.3)	6,853 (4.0)	747 (0.4)	1,046 (0.6)	12,037 (7.0)	34,684 (20.2)	2,856 (1.7)	294 (0.2)	1,046 (0.6)
Missing	**2,529,141 (20.5)**	4,628 (0.2)	38,066 (1.5)	56,793 (2.2)	68,895 (2.7)	8,394 (0.3)	72,388 (2.9)	21,707 (0.9)	25,217 (1.0)	231,798 (9.2)	559,247 (22.1)	59,091 (2.3)	10,779 (0.4)	30,421 (1.2)
**Deprivation quintile**														
1 least deprived	**2,438,403 (19.7)**	7,237 (0.3)	63,537 (2.6)	92,147 (3.8)	109,168 (4.5)	11,610 (0.5)	116,807 (4.8)	31,930 (1.3)	39,387 (1.6)	386,861 (15.9)	816,779 (33.5)	97,006 (4.0)	13,619 (0.6)	39,700 (1.6)
2	**2,405,342 (19.5)**	7,183 (0.3)	59,183 (2.5)	91,519 (3.8)	108,591 (4.5)	11,727 (0.5)	129,822 (5.4)	33,172 (1.4)	36,872 (1.5)	386,346 (16.1)	812,461 (33.8)	97,405 (4.0)	15,069 (0.6)	40,319 (1.7)
3	**2,330,927 (18.9)**	6,440 (0.3)	50,315 (2.2)	82,878 (3.6)	98,441 (4.2)	11,330 (0.5)	135,457 (5.8)	31,067 (1.3)	31,679 (1.4)	358,810 (15.4)	759,158 (32.6)	91,305 (3.9)	15,270 (0.7)	39,128 (1.7)
4	**2,512,131 (20.3)**	6,069 (0.2)	43,970 (1.8)	80,012 (3.2)	94,289 (3.8)	11,982 (0.5)	157,097 (6.3)	32,438 (1.3)	29,912 (1.2)	365,489 (14.5)	778,630 (31.0)	90,960 (3.6)	17,081 (0.7)	42,975 (1.7)
5 most deprived	**2,368,487 (19.2)**	5,524 (0.2)	38,018 (1.6)	75,068 (3.2)	83,881 (3.5)	11,895 (0.5)	163,902 (6.9)	33,808 (1.4)	27,041 (1.1)	340,040 (14.4)	728,575 (30.8)	92,713 (3.9)	20,027 (0.8)	40,884 (1.7)
Missing deprivation	**300,458 (2.5)**	839 (0.3)	5,943 (1.9)	10,167 (3.3)	11,490 (3.8)	1,798 (0.6)	18,216 (6.0)	3,858 (1.3)	4,225 (1.4)	47,854 (15.6)	107,921 (35.2)	12,457 (4.1)	1,824 (0.6)	4,905 (1.6)

*Most recent recorded blood pressure (BP) > 140/90 or patient was prescribed an antihypertensive in last 6 months. CKD = chronic kidney disease IQR = interquartile range

**Table 2:** Prevalence of mental health conditions overall and by socio-demographics within CPRD Aurum database, 2020

	All	Alcohol misuse	Anxiety	General Anxiety Disorder only	Bipolar disorder	Depression	Eating disorder	PTSD	Schizophrenia	Substance misuse
Number (%)	12,361,554	536,089 (4.34)	1,748,846 (14.15)	957,808 (7.75)	46,648 (0.38)	1,980,113 (16.02)	79,134 (0.64)	61,850 (0.50)	53,833 (0.44)	211,226 (1.71)
**Age (median, IQR)**	**39.5 (22.5–58.5)**	52.5 (39.5–63.5)	48.5 (33.5–61.5)	48.5 (34.5–60.5)	51.5 (39.5–63.5)	50.5 (37.5–62.5)	37.5 (27.5–49.5)	45.5 (34.5–55.5)	52.5 (41.5–63.5)	41.5 (32.5–52.5)
**Age Categories (years)**										
0–16	**2,215,109 (17.9)**	719 (0.03)	29,994 (1.35)	7,777 (0.35)	20 (< 0.01)	3,414 (0.15)	4,830 (0.22)	549 (0.02)	253 (0.01)	1,057 (0.050
17–30	**2,157,953 (17.5)**	55,187 (2.56)	294,323 (13.64)	154,957 (7.18)	4,062 (0.19)	238,250 (11.04)	23,430 (1.09)	9,168 (0.42)	3,553 (0.16)	41,907 (1.94)
31–40	**1,808,259 (14.6)**	79,350 (4.39)	307,744 (17.02)	174,592 (9.66)	8,040 (0.44)	332,160 (18.37)	18,328 (1.01)	13,303 (0.74)	8,608 (0.48)	52,265 (2.89)
41–50	**1,676,254 (13.6)**	100,913 (6.02)	308,789 (18.42)	180,504 (10.77)	9,474 (0.57)	381,132 (22.74)	15,454 (0.92)	14,660 (0.87)	11,346 (0.68)	51,307 (3.06)
51–60	**1,704,779 (13.8)**	126,434 (7.42)	324,712 (19.05)	188,701 (11.07)	10,554 (0.62)	427,893 (25.10)	10,681 (0.63)	13,805 (0.81)	12,952 (0.76)	35,029 (2.05)
61–70	**1,254,678 (10.2)**	94,848 (7.56)	228,910 (18.24)	126,525 (10.08)	7,298 (0.58)	298,176 (23.77)	4,406 (0.35)	6,868 (0.55)	8,753 (0.70)	14,769 (1.18)
70+	**1,544,522 (12.5)**	78,638 (5.09)	254,374 (16.47)	124,752 (8.08)	7,200 (0.47)	299,088 (19.36)	2,005 (0.13)	3,497 (0.23)	8,368 (0.54)	14,892 (0.94)
**Sex**										
Male	**6,224,433 (50.4)**	333,305 (5.4)	642,373 (10.3)	349,208 (5.6)	19,153 (0.3)	720,524 (11.6)	10,271 (0.2)	31,011 (0.5)	31,383 (0.5)	136,864 (2.2)
Female	**6,137,121 (49.6)**	202,784 (3.3)	1,106,473 (17.8)	608,600 (9.8)	27,495 (0.4)	1,259,589 (20.2)	68,863 (1.1)	30,839 (0.5)	22,450 (0.4)	74,362 (1.2)
**Ethnicity**										
White	**7,919,608 (64.1)**	432,839 (5.5)	1,335,223 (16.9)	741,373 (9.4)	36,401 (0.5)	1,519,840 (19.2)	59,086 (0.7)	41,816 (0.5)	36,622 (0.5)	153,861 (1.94)
Asian	**1,024,958 (8.3)**	25,619 (2.5)	70,796 (6.9)	37,551 (3.7)	2,568 (0.3)	79,691 (7.8)	3,354 (0.3)	4,617 (0.5)	4,661 (0.5)	11,630 (1.13)
Black	**508,581 (4.1)**	16,261 (3.2)	34,378 (6.8)	18,777 (3.7)	1,884 (0.4)	45,635 (9.0)	1,375 (0.3)	4,441 (0.9)	5,471 (1.1)	7,991 (1.57)
Mixed ethnicity	**207,281 (1.7)**	6,113 (3.0)	19,397 (9.4)	10,886 (5.3)	787 (0.4)	21,873 (10.6)	1,205 (0.6)	1,327 (0.6)	1,433 (0.7)	4,040 (1.95)
Other ethnicity	**171,985 (1.4)**	4,831 (2.8)	13,763 (8.0)	7,719 (4.5)	407 (0.2)	16,111 (9.4)	551 (0.3)	1,795 (1.0)	589 (0.3)	2,807 (1.63)
Missing	**2,529,141 (20.5)**	50,426 (2.0)	275,289 (10.9)	141,502 (5.6)	4,601 (0.2)	296,963 (11.7)	13,563 (0.5)	7,854 (0.3)	5,057 (0.2)	30,897 (1.12)
**Deprivation quintile**										
1 least deprived	**2,438,403 (19.7)**	77,062 (3.2)	326,861 (13.4)	166,450 (6.8)	7,148 (0.3)	352,608 (14.5)	16,656 (0.7)	7,607 (0.3)	5,300 (0.2)	20,346 (0.8)
2	**2,405,342 (19.5)**	93,783 (3.9)	337,197 (14.0)	177,534 (7.4)	7,924 (0.3)	371,569 (15.4)	16,057 (0.7)	9,118 (0.4)	7,034 (0.3)	27,416 (1.1)
3	**2,330,927 (18.9)**	94,271 (4.0)	321,720 (13.8)	173,302 (7.4)	8,667 (0.4)	364,235 (15.6)	15,048 (0.6)	10,729 (0.5)	9,190 (0.4)	34,626 (1.5)
4	**2,512,131 (20.3)**	122,407 (4.9)	342,762 (13.6)	193,470 (7.7)	10,066 (0.4)	396,983 (15.8)	15,009 (0.6)	14,379 (0.6)	13,769 (0.5)	51,196 (2.0)
5 most deprived	**2,368,487 (19.2)**	136,496 (5.8)	367,829 (15.5)	216,640 (9.1)	11,189 (0.5)	436,521 (18.4)	14,031 (0.6)	17,769 (0.8)	16,927 (0.7)	71,836 (3.0)
Missing deprivation	**300,458 (2.5)**	12,070 (3.9)	52,477 (17.13)	30,412 (9.9)	1,654 (0.5)	58,197 (19.0)	2,333 (0.8)	2,248 (0.7)	1,613 (0.5)	5,806 (1.9)

PTSD = post-traumatic stress disorder IQR = interquartile range

For analysis of prevalence by socio-demographic variables the adjusted odds ratios for prevalence of each condition by sex, deprivation quintile, age categories and ethnicity were calculated. These are presented in forest plots in Additional File 2.

#### Sex

Cardio-renal-metabolic conditions were more prevalent in men, except for CKD which was more prevalent in women. There was no difference in the prevalence of PTSD between men and women. Affective (depression, anxiety and bipolar) and eating disorders were more prevalent in women, whilst there were higher odds of substance and alcohol misuse and schizophrenia in men. [See Additional File 2; Supplementary Fig. 1]

#### Socio-economic status

There was a clear trend of increasing prevalence of almost all conditions with increasing socio-economic deprivation, with ORs in the order of 1.4 (aortic aneurysm) to 3.9 (substance misuse) greater in those from the most compared to the least deprived. Associations were weaker between deprivation and AF, heart valve disorders, and T1DM, and prevalence decreased with increasing deprivation for eating disorders. [See Additional File 2; Supplementary Fig. 2]

#### Age categories

There was a general trend of increasing lifetime prevalence for all cardio-renal-metabolic conditions (except for T1DM) with increasing age. There was a marked increase in prevalence of all mental health conditions after the age of 16. There was typically a gradual increase in lifetime prevalence of each mental health condition up until the age of 40–60 followed by a gradual decrease in recorded prevalence in the oldest age categories. Lower lifetime prevalence of a MH condition in those over 60 years old was most pronounced for substance abuse and PTSD. [See Additional File 2; Supplementary Fig. 3]

#### Ethnicity

There was considerable variation in the prevalence of CRM and MH conditions by ethnicity. Among those of black and Asian ethnicities diabetes, hypertension, and CKD, were more prevalent than in those of white ethnicity, whilst aortic aneurysms, AF, PVD, heart valve disorders and T1DM were less prevalent in black or Asian people.

In CPRD data, mental health conditions were typically around twice as prevalent in those of white ethnicity as in those of black or Asian ethnicity, except for PTSD and schizophrenia, which were 33% more prevalent and twice as prevalent in those of black ethnicity.

[See Additional File 2; Supplementary Fig. 4]

### Comparison of prevalence of health conditions in CPRD against literature

**Fig. 1:**
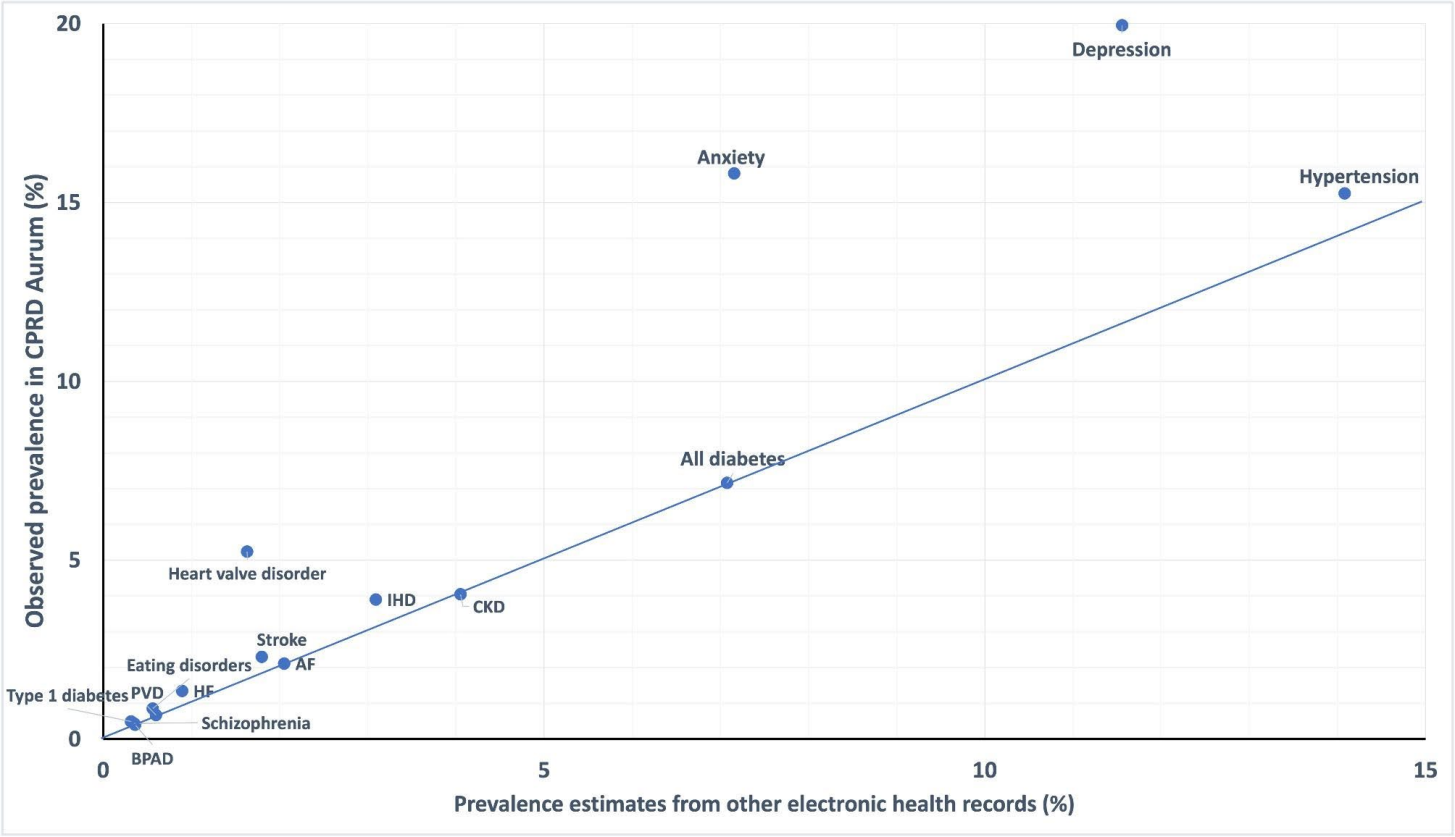
Comparison of condition prevalences in CPRD Aurum with prevalence estimates from other electronic health records. CKD = chronic kidney disease, IHD = ischaemic heart disease, AF = atrial fibrillation, HF = heart failure, PVD = peripheral vascular disease, BPAD = bipolar affective disorder

**Fig. 2:**
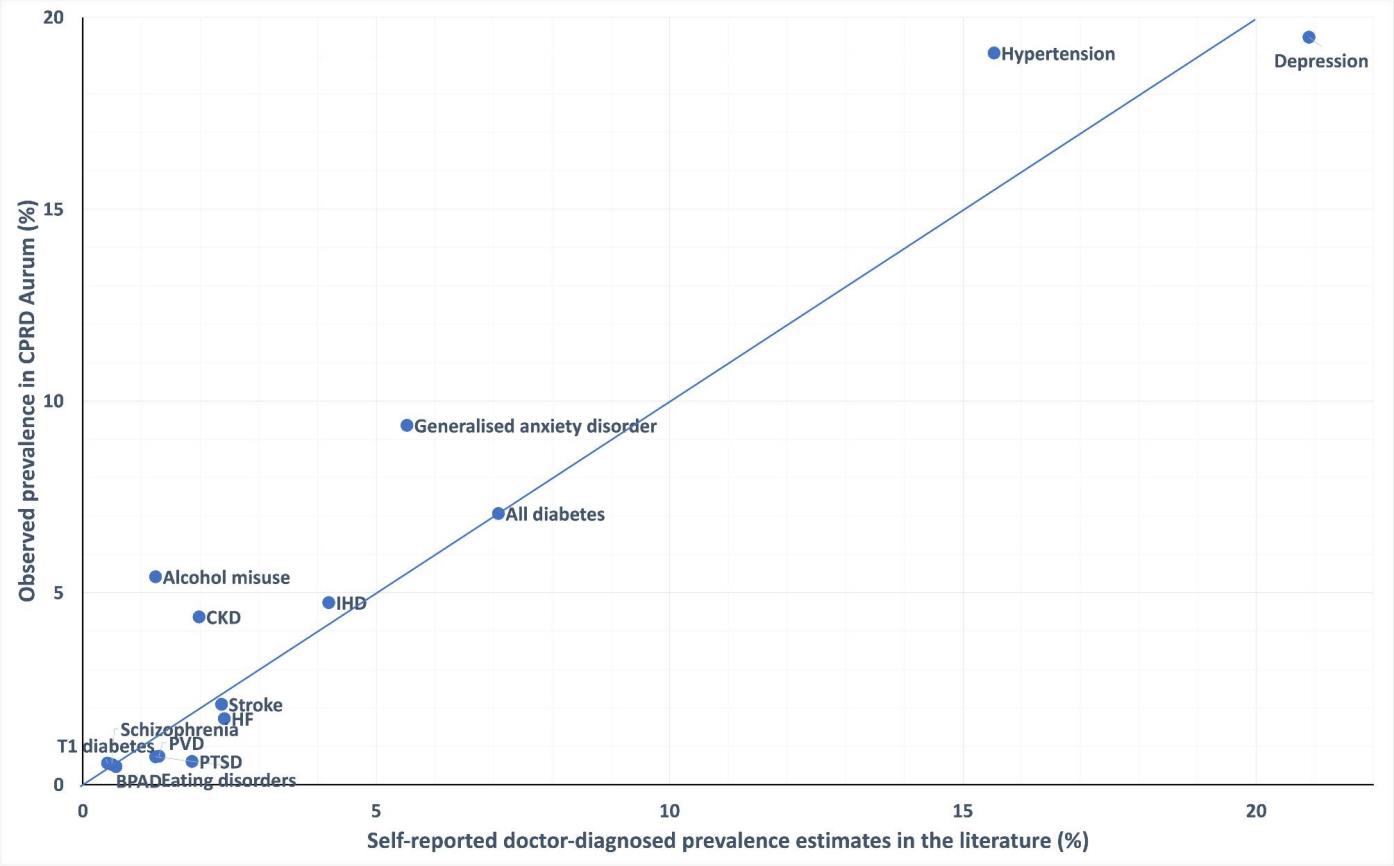
Comparison of condition prevalences in CPRD Aurum with self-reported doctor-diagnosed prevalence estimates from the literature. CKD = chronic kidney disease, IHD = ischaemic heart disease, HF = heart failure, PVD = peripheral vascular disease, BPAD = bipolar affective disorder, T1 diabetes = type 1 diabetes, PTSD = post-traumatic stress disorder

**Fig. 3:**
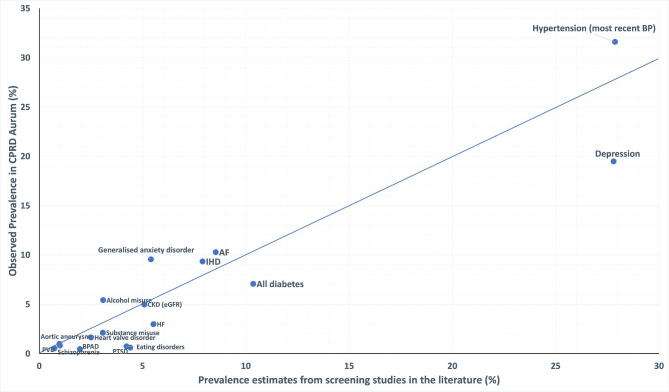
Comparison of condition prevalences in CPRD Aurum with screening study prevalence estimates from the literature. BP = blood pressure, CKD = chronic kidney disease, IHD = ischaemic heart disease, AF = Atrial fibrillation, HF = heart failure, PVD = peripheral vascular disease, BPAD = bipolar affective disorder, PTSD = post-traumatic stress disorder

Figures 1, 2 and 3 compare the prevalence estimates from the literature within other UK primary care EHRs (Fig. 1), surveys of self-reports of doctor-diagnosed conditions (Fig. 2) and screening studies (Fig. 3), against the prevalence of each condition in an age matched population within CPRD Aurum. Prevalence estimates from the literature, with the data sources and methods of data collection are reported in Additional File 1; Tables 1, 2 and 3.

### Prevalence in UK primary care EHRs

Figure 1 shows that for 5/10 CRM conditions, the prevalence in CPRD Aurum was similar to (< 20% difference relative to) available prevalence estimates for age-matched populations in QOF and other UK primary care EHRs [18]. However, the prevalence of heart valve disorders in CPRD Aurum in 65–95-year-olds (5.2% (95%CI 5.2–5.3%)) was more than double the prevalence reported in age-matched patients in THIN data (1.6% (95%CI 1.6–1.7%) [28]. The prevalences of IHD, T1DM, stroke and HF were between 20 and 55% higher in CPRD Aurum than in other UK primary care EHRs [18, 29–31].

The prevalence of bipolar disorder in CPRD Aurum (0.4% (95%CI 0.4–0.4%)), was similar to (< 20% higher than) the prevalence estimate in the IQVIA Medical Research Database (IMRD) in 2018 (0.4% (95%CI 0.4–0.4%)) [32]. The prevalence of eating disorders and schizophrenia in CPRD Aurum were 20% and 34% higher respectively than prevalence estimates in CPRD Gold [33–35]. For depression and anxiety the age-matched prevalence in CPRD Aurum was around twice as high as in QOF and THIN data [18, 36].

### Self-reported doctor-diagnosed prevalence

Figure 2 shows the prevalence of stroke, diabetes, and IHD in CPRD Aurum were similar to (< 20% difference relative to) self-reported doctor-diagnosed prevalence estimates in HSE [23, 37]. However, prevalence of CKD in over 16 year olds was more than twice as high in CPRD Aurum (4.4% (95%CI 4.4–4.4%) than in HSE data (2.0% (95%CI 1.6–2.4%)) [38]. Prevalence of T1DM and hypertension in CPRD Aurum were 23% and 34% higher than were reported by the National Diabetes Audit and HSE respectively [23, 30]. Prevalence of PVD in CPRD Aurum was 43% lower compared with the prevalence reported in UK Biobank [39].

The prevalence of depression, schizophrenia and bipolar disorder in CPRD Aurum in over 16 year olds closely matched (< 20% relative difference to) those reported in HSE and APMS [14, 37]. The prevalence of eating disorders in CPRD Aurum was 41% lower reported in the HSE, [34]whilst for generalised anxiety disorder prevalence was 69% higher in CPRD Aurum than in the HSE [37]. Prevalence of alcohol misuse was three times higher in CPRD Aurum (5.4% (95%CI 5.4–5.4%)) than in HSE (1.2% (95%CI 1.0-1.5%)) [37]. However, prevalence of PTSD in CPRD Aurum (0.6% (95%CI 0.6–0.7%) was three times lower than that reported by HSE (1.9% (95%CI 1.5–2.2%)) [37].

### Screen-detected prevalence

Figure 3 shows that for aortic aneurysms, CKD, IHD, AF and PVD, the prevalence estimates reported in CPRD Aurum matched (< 20% difference relative to) estimates of screen-detected prevalence in the same age groups in the literature [29, 38, 40–42]. For diabetes, hypertension, heart failure, and heart valve disorder the prevalence estimates in CPRD Aurum were around a third lower than in screening studies [23, 43, 44].

For substance misuse disorder, depression, and schizophrenia the prevalence estimates in CPRD Aurum were around 30% lower than in the APMS (2014) [14]. For generalised anxiety disorder and alcohol misuse disorder, the prevalence in CPRD Aurum were around 80% higher than in the European Study of the Epidemiology of Mental Disorders and APMS respectively [14, 45]. However, for eating disorders, bipolar disorder and PTSD, prevalence reported in the APMS and HSE were 4–6 times higher than in CPRD Aurum [14, 33].

### Biomarkers for hypertension and CKD

When defined by use of antihypertensive medication or most recent blood pressure reading > 140/90mmHg (to match methodology in HSE) the prevalence of hypertension in CPRD Aurum in over 16-year-olds was 31.6% (95%CI 31.6–31.6%)), which was almost twice as high as the prevalence when defined by using clinical codes (19.1% (95%CI 19.0- 19.1%)). However, as shown in Fig. 3 it was similar to (< 20% difference relative to) the HSE screen-detected prevalence estimate (27.9% (95%CI 26.5–29.2%)) [23].

Prevalence of CKD in CPRD in over 16-year-olds was similar (< 20% relative difference) when measured using the most recent eGFR < 60ml/min/1.73 m² (to match methodology in HSE) (5.0% (95%CI 5.0–5.0%)) to both the prevalence in CPRD estimated using clinical codes (4.4% (95%CI 4.4–4.4%)) and the screen-detected prevalence in HSE (5.1% (95%CI 4.4–5.8%)) [38].

## Discussion

### Main findings

This was a comprehensive analysis of the prevalence of cardio-renal-metabolic (CRM) and mental health (MH) conditions in 12 million patients in a primary care electronic health records (EHRs) database. There was a high burden of depression, anxiety, and hypertension across the population. As expected, most conditions reported in EHRs were increasingly prevalent with increasing deprivation and age, although mental health conditions were potentially under-represented in children. Most CRM conditions, schizophrenia and substance misuse were more prevalent in men, whilst anxiety, depression, bipolar and eating disorders were more common in women. Hypertension and diabetes were twice as prevalent in black patients compared with white patients and diabetes was three times as common in Asian patients. However, black and Asian patients generally had lower recorded prevalences of cardiovascular disease (aortic aneurysms, AF, PVD, HF, heart valve disorder, IHD, stroke) than white patients. Mental health conditions were reported twice as frequently in those of white ethnicity as in those of black or Asian ethnicity in EHRs, except for PTSD and schizophrenia, which were 33% more prevalent and twice as prevalent in those of black ethnicity respectively.

Estimates for prevalence of most clinically detected CRM conditions, as well as depression, anxiety, bipolar disorder, and schizophrenia in the EHR database were broadly similar or greater than the self-reported doctor-diagnosed prevalence reported in the Health Survey for England (HSE) and Adult Psychiatric Morbidity Survey (APMS). This suggests these conditions are well represented in EHRs. However, there were sizable differences in the prevalence of hypertension, diabetes, and depression in the EHR compared to other prevalence estimates from studies screening for these conditions. Screen-detected prevalence estimates for PTSD, bipolar disorder and eating disorders were 4–6 times higher than prevalence of these conditions in primary care EHR records, potentially reflecting a significant burden of underdiagnosed or less well documented MH morbidity.

### Comparisons with other literature

In EHR the risk factors for cardiovascular disease (i.e., hypertension and diabetes) were more prevalent in black and Asian people than white people, but paradoxically this was not typically matched by higher prevalence of cardiovascular disease itself (i.e., PVD, aortic aneurysms, stroke, IHD). This has also been reported in other cohort studies analysing variation in prevalence of aortic aneurysms and peripheral artery disease by ethnicity [46, 47]. We found that AF was recorded twice as frequently in white patients compared with black and Asian patients. A previous cross-sectional analysis also found lower prevalence of AF recorded in African American patients’ records compared with white American patients, but no difference in prevalence with systematic unbiased testing [48]. Potential explanations have included differential uptake of screening in the case of aortic aneurysms, and under-diagnosis due to language barriers or lower-health literacy in Asian people regarding PVD symptoms [20].

These disparities may also reflect the higher premature death rate from IHD in Asian people compared to white people, thus susceptible Asian people do not survive long enough to.

develop symptoms of PVD [20]. Additionally, although these analyses of variation in prevalence by ethnicity are adjusted for age, given the strength of the association between age and cardiovascular diseases, the lower prevalence of cardiovascular disease in those of black and Asian ethnicity could reflect that in this database these populations were on average significantly younger than those of white ethnicity.

In mental health conditions there was typically significant reduction in prevalence in the over 70-year-olds compared with those aged 40–50, which may reflect earlier mortality for those diagnosed with these conditions at younger ages [49]. Reduced prevalence in the oldest adults is especially notable in eating disorders (see Additional File 2; Supplementary Fig. 3), which is the MH condition with highest mortality rate [50]. In the analyses of prevalence conditions by socio-demographic factors; it is important to note that those who have died before the index date were excluded from the sample so those with non-fatal disease may be over-represented in the survivors.

Prevalence of MH conditions recorded in the primary care EHR was comparatively very low in children. Depression was recorded 40 times more frequently in 17–30 year-olds compared with under-16 year-olds. The latest Mental Health of Children and Young People in England survey found that one in six people aged 6–16 years had a “probable” MH condition [51]. However, this reflects a wide range of mental health symptoms from mood and anxiety to attention and hyperactivity, rather than specific diagnoses. Nevertheless, there is likely to be considerable under-representation of the true prevalence of MH conditions in children in EHRs. Qualitative research suggests that whilst parents and children do not always report mental health symptoms to GPs, [52] in turn GPs report feeling ill-equipped to diagnose MH conditions in children, and there are considerable challenges in accessing child and adolescent mental health specialists [52–54].

The gap between screen-detected prevalence and primary care EHR prevalence was more apparent for MH conditions than for CRM conditions, notably for depression, bipolar disorder, eating disorders and PTSD. Financial incentives for accurate coding of certain conditions may have impacted the accuracy of recording diagnoses in EHR. All but one (aortic aneurysms) of the CRM conditions are included in QOF, which financially incentivises practices to have accurate disease coding, whilst eating disorders and PTSD are not included in QOF [18]. Longitudinal analysis of atrial fibrillation coding in UK EHRs suggests that the introduction of QOF did lead to practices refining the diagnostic coding for this condition [55].

PTSD had the most notable discrepancies between both screen-detected prevalence and self-reported doctor-diagnosed prevalence compared with prevalence in the EHR, which suggests that this condition may be especially under-recognised and under-diagnosed. PTSD is typically diagnosed in secondary care and case-detection within primary care EHR is also dependent on accurate transfer of information between primary and secondary care. Studies exploring the accuracy of stroke and cancer diagnoses in primary care EHR, have shown that between 10 and 40% of these diagnoses in hospital records are missing in primary care EHR [14]. Many people with symptoms of common MH conditions do not present to primary or secondary care [13]. However, self-reported screening questionnaires also consistently overestimate the prevalence of MH conditions in epidemiological studies, [56] thus CPRD Aurum and other EHR databases may be more reliable for case-detection of these conditions. Results from the SAIL EHR databank, showed that ten-year prevalence of depression and/or anxiety was 16.2% and of anxiety/depression symptom codes was 21.4% which is similar to our estimates (16.0% had depression (95%CI 16.0–16.0%) [57].

Women had double the rates of reported depression and anxiety compared with men in the primary care EHR. However, in the AMPS survey screening for symptoms of depression and anxiety prevalence of these conditions is only around 25–50% higher in women [13]. In the EHR, depression and anxiety were three times as common in those of white ethnicity compared with those of black or Asian ethnicity. However, in the AMPS survey, symptoms of depression and anxiety were more common in people of black and Asian ethnicity [13]. Like previous studies, we found that black people were twice as likely to be diagnosed with schizophrenia as other ethnicities [13]. Research in this area is limited by small sample sizes. However, it is recognised that there are considerable barriers to accessing mental healthcare for people from black and minority ethnic communities, which may lead to under-diagnosis in primary care [58]. These disparities between screening prevalence and prevalence of mental health conditions in EHR likely reflect patterns of help-seeking behaviour and barriers to access, which are influenced by both gender and ethnicity [58, 59].

There was also an overall gap between screen-detected prevalence in HSE and CPRD Aurum prevalence for diabetes and hypertension, whilst doctor-diagnosed prevalence estimates were similar [23]. However, it is important to note that the methods used for screening in HSE are not diagnostic, for example, a single raised HbA1c measurement was used to estimate the prevalence of diabetes, whereas clinical guidelines state that two raised HbA1c measurements are required to confirm the diagnosis.

Replicating the screening methods used in HSE with clinical biomarkers such as blood creatinine and blood pressure produced a similar prevalence rate of hypertension and CKD [23]. These biomarkers may be useful for some studies looking at short term outcomes. A previous study in CPRD Gold found that clinical codes underestimate the prevalence of CKD and concluded that a combination of codes and test results is most appropriate to detect CKD [60]. However, for studies investigating multimorbidity and detection of disease accumulation over several years, clinical codes are more likely to be more specific and most reflective of long-term conditions.

The prevalence of all CRM and MH conditions in CPRD Aurum typically ranged from 5 to 50% higher than prevalence rates reported in other UK primary care EHR databases (predominantly QOF data). Our codelists were more comprehensive than QOF codelists; for example, the codelists for heart failure and depression included more codes related to interventions, abnormal test results, disease monitoring, and referral to secondary care services. In both these conditions the prevalence estimates in CPRD Aurum were similar to the self-reported doctor-diagnosed prevalence estimates. Therefore, our codelists may be more sensitive but less specific than QOF codelists.

A diagnosis of anxiety was more prevalent in CPRD Aurum data (15.8% (95%CI 15.8–15.8%)) in comparison with a previous analysis of THIN data (7.2% (95%CI 7.1–7.2%)) [36]. However, the THIN analysis reported prevalence of anxiety codes entered between 2002 and 2004 only, whereas we included any case prior to 2020. Doctor-diagnosed prevalence of generalised anxiety disorder was also higher in CPRD Aurum (9.4% 95%CI 9.4–9.4%)) compared with self-reported doctor-diagnosed generalised anxiety in HSE (5.5% (95%CI 4.9–6.1%)) [37]. The most frequently used code within our anxiety codelist by some margin was “Anxiety with depression”, reflecting the established overlap between these two conditions.

As in previous studies, the prevalence of all conditions increased with increasing socio-economic deprivation (with the exception of eating disorders) [61]. A recent systematic review showed no consistent pattern of association between socio-economic status and eating disorders, but that historically those in more affluent groups were more likely to access diagnosis and treatment, which may explain the inverse association between social deprivation and eating disorders [62].

The prevalence of alcohol misuse in CPRD Aurum in over 16-year-olds (5.4% (95%CI 5.4–5.4%)) was considerably higher than HSE reports of both self-reported doctor-diagnosed alcohol misuse (1.2% (95%CI 1.0-1.5%)) and the screen-detected prevalence of alcohol misuse in the same age group (3.1% (95%CI 2.7–3.5%)). Participants may potentially under-report their true drinking practices in surveys, whilst GPs may be entering clinical codes for alcohol misuse but not conveying the extent of their concerns to patients [63]. On the other hand, substance misuse appears to be under-diagnosed in CPRD Aurum compared with self-reported substance misuse. The prevalence reported in CPRD Aurum 2.1% (95%CI 2.1–2.1%) was lower than the screen-detected prevalence of drug dependence in APMS analysis 3.1% (95%CI 2.7–3.5%), which is in keeping with findings from other studies [64].

### Strengths and limitations

This CPRD Aurum database contains EHR from over 12 million patients reflecting a nationally representative sample of the UK population in terms of geographic spread, deprivation, age, and gender [7]. For half of the 18 conditions (almost all the CRM conditions) primary care clinicians are financially incentivised via the QOF system since 2004 to accurately record diagnosis codes in EHRs.

Our codelists for identifying conditions within CPRD Aurum were created using a rigorous and systematic process by a team of experienced clinicians, building on a strong foundation of previous research using clinical codes in EHRs. Our findings demonstrate that these codelists appear to have high sensitivity to detect the majority of CRM and MH conditions within EHRs.

The literature review was more pragmatic than a systematic review methodology as it would not have been feasible to do a systematic review for each of the 18 conditions. However, the majority of the comparisons are from the latest official UK government commissioned studies or audits of disease prevalence (e.g., QOF, HSE, APMS, National Diabetes Audit, etc.) [30]. Comparisons with studies reliant on self-reported health status (e.g., HSE) are subject to response bias which may have influenced their findings.

An important limitation is that the prevalences we report are lifetime prevalences, thus conditions that have resolved will still be captured in our results. This was done for comparison with the analysis periods of the comparator data sources. Many of the included conditions are likely to be lifelong conditions (e.g., type 1 diabetes or heart failure). However, others such as depression or anxiety may later resolve or follow a relapsing-remitting course, rather than having persisting symptoms. Therefore, the duration of the data collection period significantly affects the reported prevalence of these types of conditions.

For pragmatic reasons, only age (and sex in the case of aortic aneurysms) was used to stratify CPRD Aurum data to make comparisons with prevalence estimates from the literature. Where disease prevalence has changed over time, especially given the ageing population, there can be far less certainty in the comparisons with prevalence estimates from less recent studies in the literature. Caution should be taken in analysis of prevalence of conditions by ethnicity, given that these categories aggregate together very diverse communities and ranges of cultural practices and countries of ethnic origin. Where researchers wish to examine specific conditions or sub-populations in more depth or wish to understand prevalence within a specific sub-population these factors may need to be explored in greater detail.

### Implications for policy and practice

Primary care EHR data are a reliable source for clinically diagnosed cases of most cardio-renal-metabolic (CRM) conditions and for depression, bipolar disorder, and schizophrenia. Caution should be taken in interpreting analyses of anxiety disorders using primary care EHR data as the prevalence may be over-reported, whereas cases of PTSD and eating disorders may be under-reported. Policymakers should explore whether incentivising accurate coding for more MH conditions, for example through QOF (in the UK), could improve reporting quality [18]. Policymakers may also wish to consider how both public awareness and primary care and mental health services can be configured to improve case-detection of these more neglected MH conditions, especially in men and children and those of black or Asian ethnicity. Healthcare providers should be encouraged to adopt culturally sensitive practices to ensure that minority populations receive adequate mental health care and support.

We found almost 40% of patients on anti-hypertensive medications or whose latest recorded blood pressure was greater than 140/90mmHg did not have a clinical code for hypertension in their EHR. This is in keeping with other studies that have demonstrated a significant burden of hypertension that is not well documented or acted on in primary care despite financial incentivisation [65]. Practices should consider implementing more robust follow-up systems once hypertension is initially detected. Policymakers should be aware that longer consultation times and higher GP to patient ratios are associated with better hypertension case-detection and management, especially in more deprived areas [65, 66].

### Implications for future research

The variation in prevalence of conditions by sociodemographic characteristics, especially sex and ethnicity, warrants further exploration to understand the relative contribution of genetics, and lifestyle, socio-cultural and healthcare-related factors in these disparities. This requires both longitudinal analyses, stratified by these demographic subgroups, to understand how these factors mediate risk of CRM and MH conditions, and qualitative research exploring barriers to accurate case-detection at both a patient and practice level (e.g., staffing ratios, funding, and continuity of care).

For future research using EHRs, additional algorithms may be used to adjust sensitivity or specificity of a codelist for case-detection of these conditions, depending on the purpose of the analysis. These might include use of prescription data, or codes for symptoms or referrals (instead of diagnoses), and use of clinical biomarkers such as blood test results. Future research could also explore the prevalence and demographic variation of other common chronic conditions in this database, such as cancer, respiratory conditions, and autoimmune diseases.

## Conclusion

Most clinically diagnosed conditions appeared to be well represented in primary care records. However, we found important variations in prevalence by demographic characteristics, which may reflect true variation in prevalence or systematic differences in likelihood of both presentation to healthcare professionals and of being diagnosed with these conditions. Primary care data may underrepresent the prevalence of undiagnosed conditions particularly in mental health.

### Electronic supplementary material

Below is the link to the electronic supplementary material.

Supplementary Material 1

Supplementary Material 2

## Data Availability

Access to anonymized patient data from CPRD Aurum is subject to a data sharing agreement containing detailed terms and conditions of use following protocol approval from the MHRA Independent Scientific Advisory Committee (CPRD Study ID 22_001903). This study-specific analysable dataset is therefore not publicly available but can be requested from the corresponding author at K.Nirantharan@bham.ac.uk subject to research data governance approvals. Details about Independent Scientific Advisory Committee applications and data costs are available on the CPRD website (cprd.com). All methods were carried out in accordance with relevant guidelines and regulations. The codelists used to define the disease definitions and perform the analysis are publicly available at https://github.com/THINKINGGroup/phenotypes. and https://github.com/CPRDAurumPrevalenceAnalysis/.

## Abbreviations

AF: atrial fibrillation
APMS: Adult Psychiatric Morbidity Survey
BP: blood pressure
BPAD: bipolar affective disorder
CKD: chronic kidney disease
CPRD: Clinical Practice Research Datalink
CT: computed tomography
CRM: cardiovascular, renal, and metabolic conditions
DExtER: Data Extraction for Epidemiological Research
eGFR: estimated glomerular filtration rate
HER: electronic health records
GP: general practitioner
Hba1c: glycosylated haemoglobin
HSE: Health Survey for England
IHD: ischaemic heart disease
IMD: index of multiple deprivation
IMRD: IQVIA Medical Research Database
IQR: interquartile range
MH: mental health conditions
PTSD: post traumatic stress disorder
PVD: peripheral vascular disease
QOF: Quality and Outcomes Framework
THIN: The Health Improvement Network
T1DM: type 1 diabetes mellitus
T2DM: type 2 diabetes mellitus
UK: United Kingdom
USA: United States of America
