# Supplementary material for: Prevalence and demographic variation of cardiovascular, renal, metabolic, and mental health conditions in 12 million english primary care records
Source: BMC Med Inform Decis Mak. 2023 Oct 16;23:220. doi: 10.1186/s12911-023-02296-z (PMC10580600; doi:10.1186/s12911-023-02296-z)
Supplement: Supplementary file 1 — Supplementary Material 1 [file 12911_2023_2296_MOESM1_ESM.docx]

***Sex***

*Supplementary Figure 1: Odds ratio of lifetime prevalence of each CRM and MH condition in men compared with women in CPRD Aurum, 2020*

**

**More common in women More common in men**

*Adjusted for age, ethnicity, and socio-economic status*

***Socio-economic status***

*Supplementary Figure 2: relative odds of lifetime prevalence of each CRM and MH*
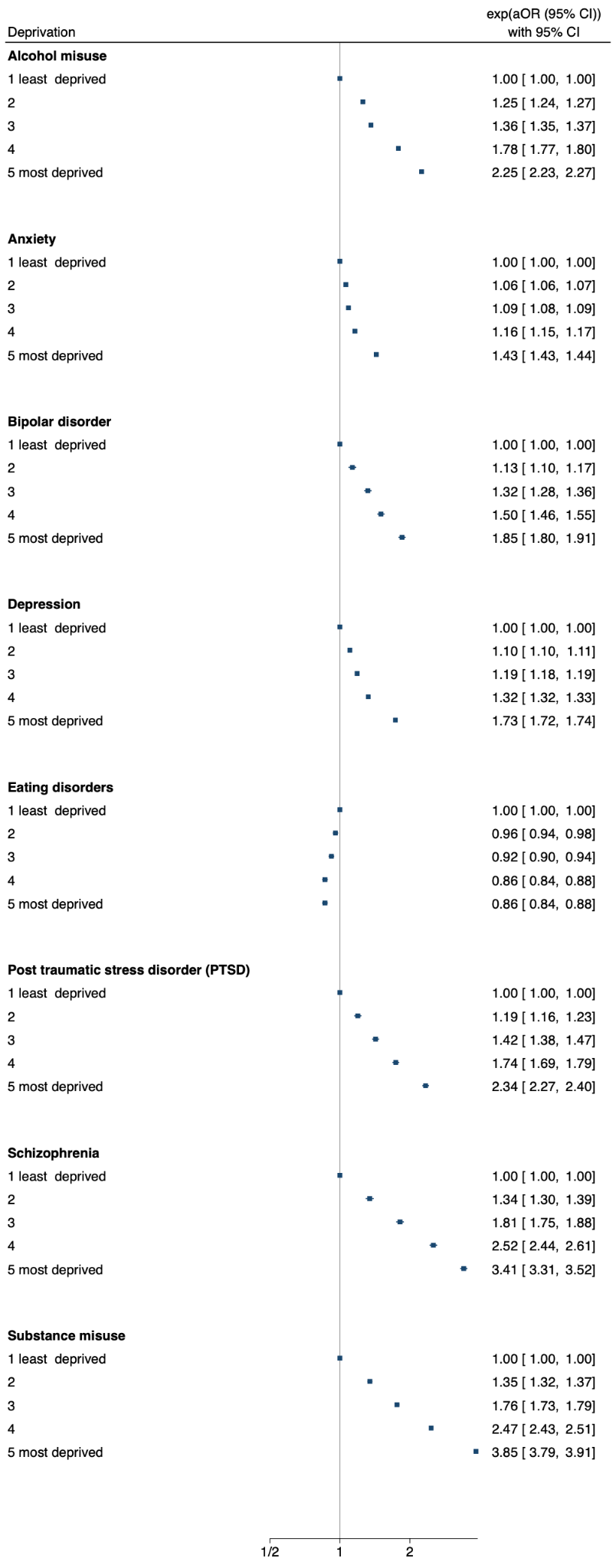
 *condition by deprivation quintile*


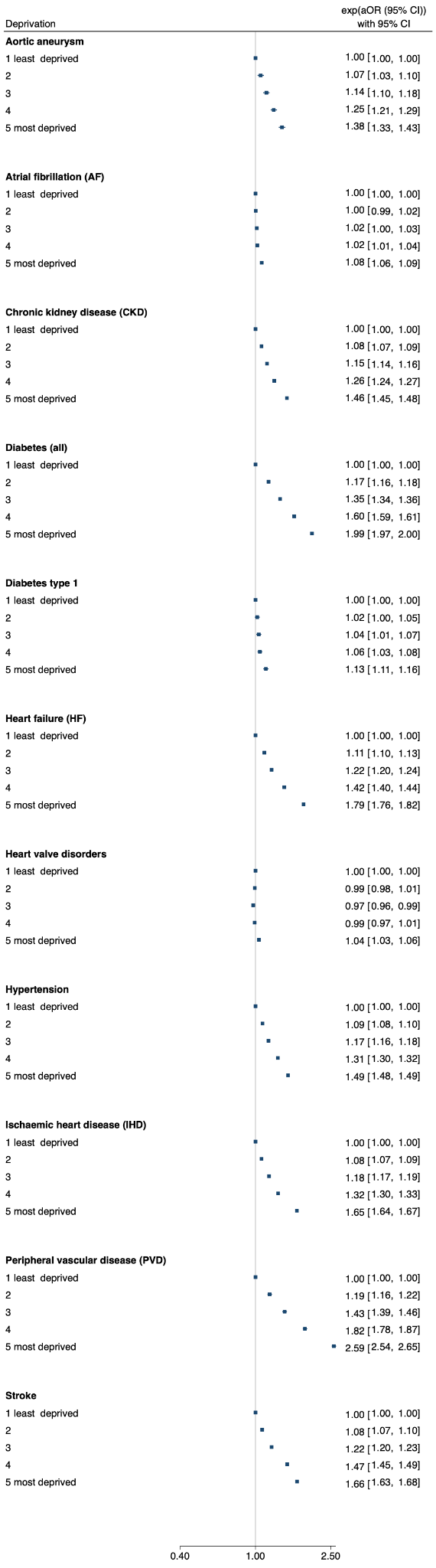


*Adjusted for age, sex, and ethnicity*

**Age categories**

*Supplementary Figure 3: relative odds of lifetime prevalence of CRM and MH conditions by age category with 41–50-year-olds as the reference category*


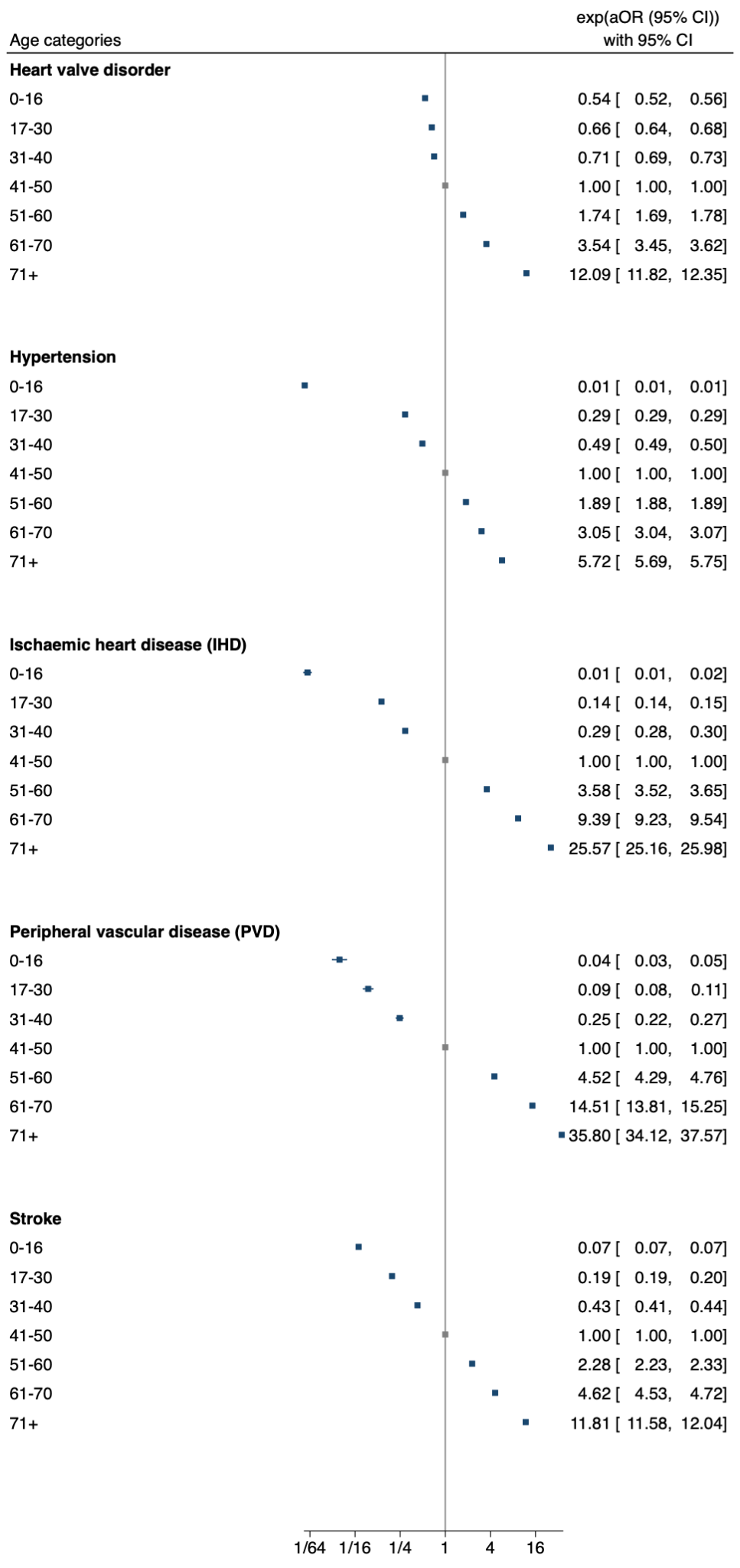

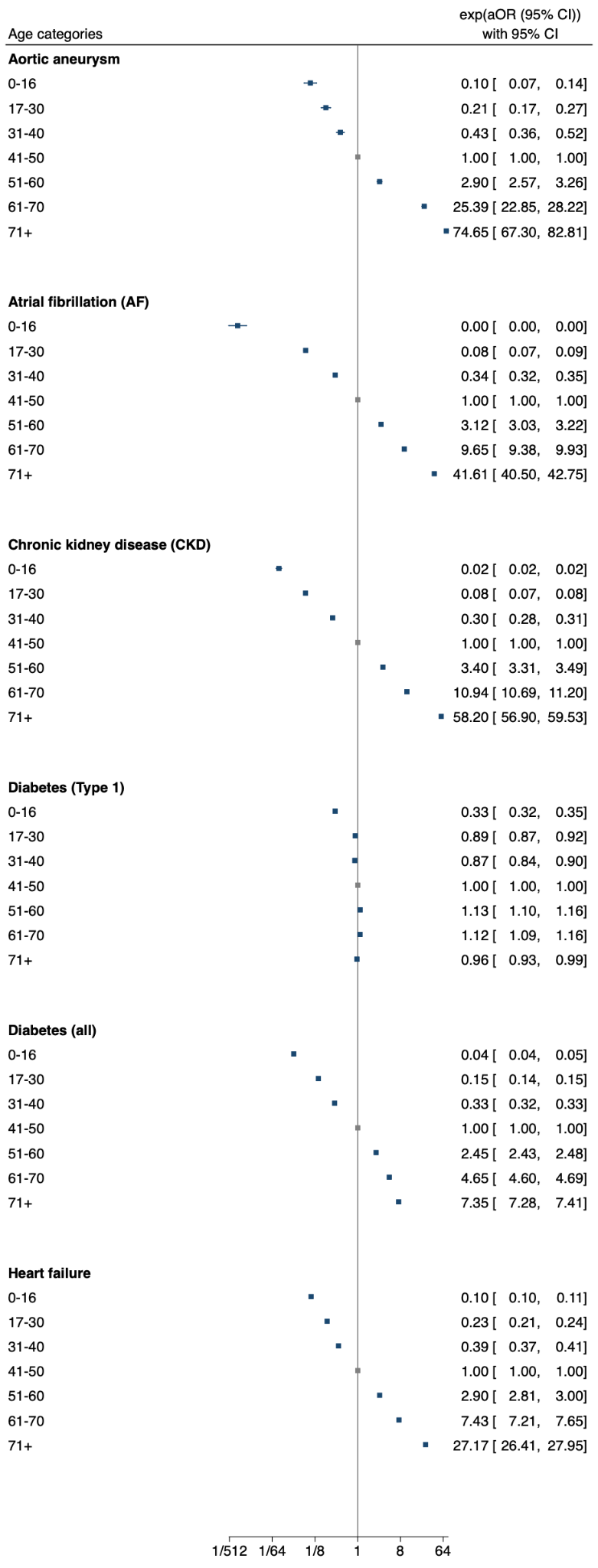


**Ethnicity**


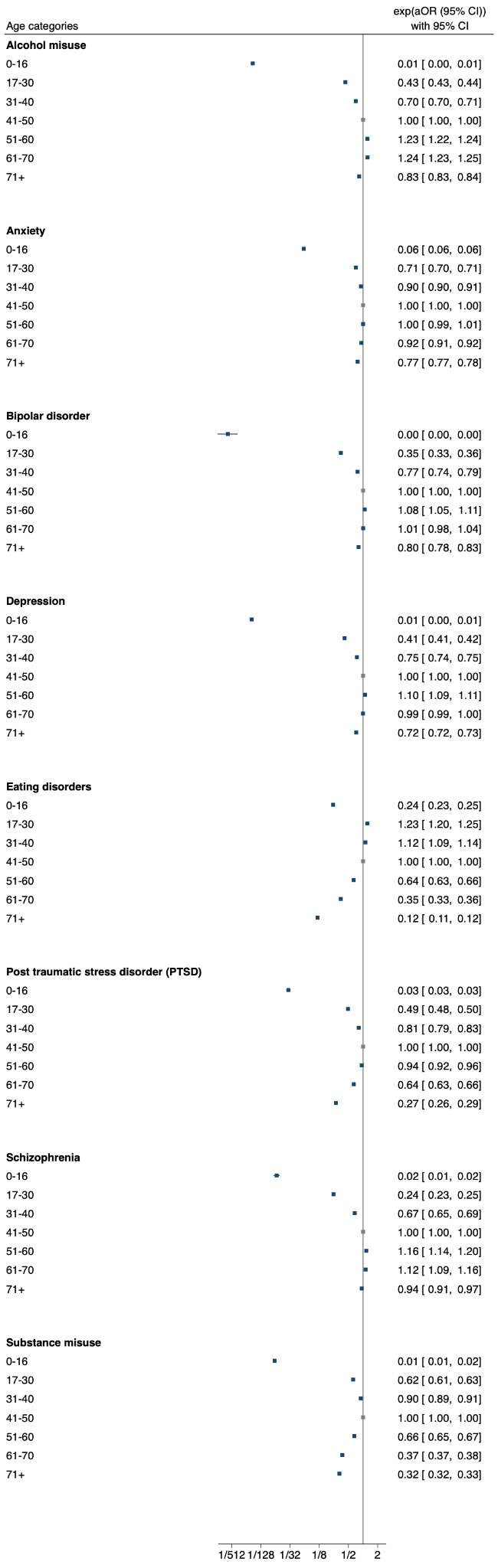


*Adjusted for sex, ethnicity,*

*and socio-economic status.*

**Ethnicity**


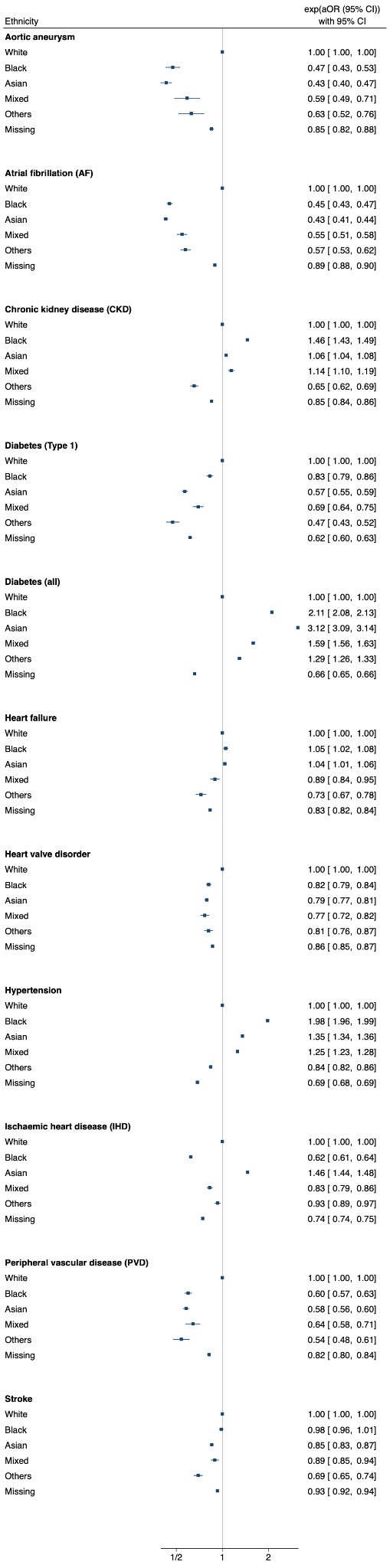
*Supplementary Figure 4: relative odds of lifetime prevalence each CRM and MH condition by ethnicity*
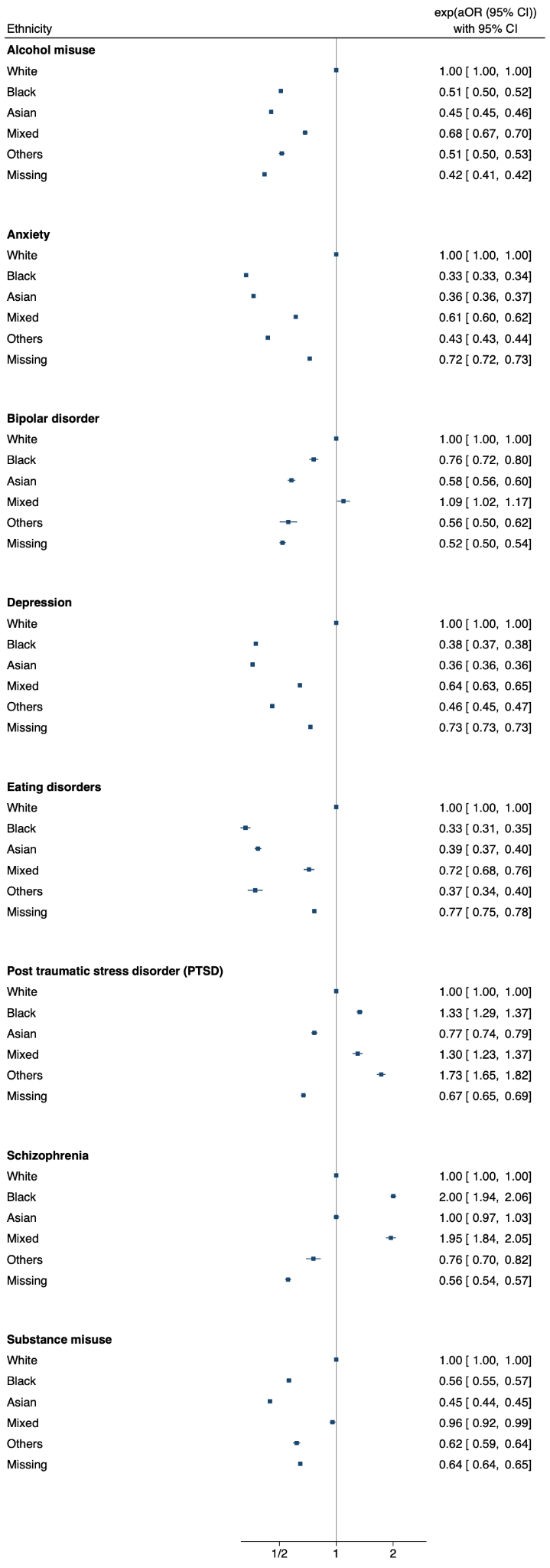


*Adjusted for age, sex, and socio-economic status*
