# Supplementary material for: Prevalence and demographic variation of cardiovascular, renal, metabolic, and mental health conditions in 12 million english primary care records
Source: BMC Med Inform Decis Mak. 2023 Oct 16;23:220. doi: 10.1186/s12911-023-02296-z (PMC10580600; doi:10.1186/s12911-023-02296-z)
Supplement: Supplementary file 2 — Supplementary Material 2 [file 12911_2023_2296_MOESM2_ESM.docx]

**Additional file 1**

**Search strategy for the literature review**

A pragmatic approach was used to identify relevant sources for each condition; where available, prevalence statistics reported within Public Health England fingertips resources, [[24]](https://paperpile.com/c/9wtXWX/lVDR) NHS Digital resources, [[25]](https://paperpile.com/c/9wtXWX/q6Zh) and NICE Clinical Knowledge Summaries were used.[[25]](https://paperpile.com/c/9wtXWX/UgiM) Where available data from QOF reporting, Health Survey for England (HSE) and Adult Psychiatric Morbidity Survey (APMS) were used as the comparator.[14,23]

1. **UK primary care electronic records prevalence**

2019/2020 QOF data was the most recent data that was reported routinely prior to the SARS-CoV-2 pandemic.[18] If QOF data were not available, PubMed and Google scholar databases were searched using the search strategy “condition name” AND “prevalence” OR “epidemiology”, AND known UK based primary care EHR databases “THIN” OR “IMRD” OR “CPRD” OR “SAIL” OR “GPRD’.

1. **Self-reported doctor-diagnosed prevalence**

The NHS Digital search engine was used to identify these studies. Where available, data from the Health Survey for England (HSE) and Adult Psychiatric Morbidity Survey (APMS) was selected. [14,23] If no relevant data were available from these sources, published UK Biobank studies were reviewed. UK Biobank is a large prospective cohort of half a million residents who had information about their health conditions collected through participant interviews (among other methods).[29] Finally, PubMed and Google scholar databases were searched for cross-sectional and longitudinal studies by “condition name” AND “prevalence” OR “epidemiology”.

1. **Screen-detected prevalence**

Where available, data from the HSE or APMS were used if these studies had administered a symptom screening questionnaire (especially in the case of mental health conditions) or an established diagnostic test (e.g. blood test for diabetes or CKD) to detect cases of each condition.[14,23] If data were not available from these sources, PubMed and Google scholar databases were searched using the search strategy “condition name” AND “screening” AND “prevalence” OR “epidemiology”.

**Sources included from literature review**

**Studies reporting disease prevalence within UK electronic healthcare records (EHR)**

Table 1 describes the data sources for the estimates of UK EHR prevalence for each condition. QOF reports were available for eight of ten CRM conditions and one of eight MH conditions. [[18]](https://paperpile.com/c/9wtXWX/Oco9) Two cross-sectional studies were identified that used CPRD Gold data (for eating disorders and schizophrenia), [[30,31]](https://paperpile.com/c/9wtXWX/Wsac+gqcW) and three cross-sectional studies that used IMRD/THIN data (anxiety, bipolar disorder and T1DM).[26,30,61] One cohort study using THIN data was identified which reported a prevalence of heart valve disorders.[28] No prevalence estimates within UK EHRs were identified for abdominal aneurysms, PTSD or drug/alcohol misuse.

**Studies reporting doctor-diagnosed prevalence of disease**

Table 2 describes the data sources for estimates of doctor-diagnosed prevalence for each condition. Three large nationally representative cross-sectional studies were identified with relevant population prevalence estimates in adults. The Health Survey for England (HSE) provided prevalence estimates for 11/18 conditions; interviewers asked a representative sample of almost 8,000 over 16-year-olds living in English households whether a doctor had diagnosed them with each specified healthcare condition. [[23]](https://paperpile.com/c/9wtXWX/JRDl) The Adult Psychiatric Morbidity Survey (APMS) provided a prevalence estimate for depression. [[14]](https://paperpile.com/c/9wtXWX/3CO0) This was also a household survey, conducted in 2014, using similar methods to HSE, which was designed to determine the prevalence of MH conditions in England. HSE did not contain prevalence data regarding heart failure. However, the National Health and Nutrition Examination Survey (NHANES) used this methodology to collect cross-sectional data on prevalence of heart failure in a US sample of 14,986 people in 2017-20.[[36]](https://paperpile.com/c/9wtXWX/2IRV)

One cohort study using UK Biobank data was identified that had collected self-reported diagnoses (and matched hospital records data) to determine cases of peripheral vascular disease at baseline. [39] A cross-sectional prevalence estimate was calculated using reported data. No doctor-diagnosed prevalence estimates were identified for aortic aneurysms, AF, or heart valve disorders.

**Studies reporting screen-detected prevalence of disease**

Table 3 describes the data sources for estimates of screen-detected prevalence for each condition. HSE and APMS explored population prevalence of 11/18 conditions in over 16-year-olds. [14,23] These studies used clinical biomarkers (including blood tests and blood pressure) and symptom questionnaires/ diagnostic interviews in a representative sample of the UK population.

For the remaining conditions, three observational studies were identified where representative samples of the population had been screened for specific conditions as well as one randomised controlled trial of a screening programme. In 2001 a community-based cohort study of 706 over 45-year-olds used echocardiography to assess prevalence of HF in a randomly selected sample of the population. [[43]](https://paperpile.com/c/9wtXWX/0St7) Public Health England’s Modelled Prevalence Estimate of IHD prevalence also largely used data from a cohort study; self-reported symptoms of angina and diagnosis of IHD in 55–79-year-olds in England in the Whitehall II cohort study. [40] Other studies have investigated the prevalence of coronary atherosclerosis on echocardiograms of asymptomatic people, although not in the UK population. [[64]](https://paperpile.com/c/9wtXWX/kuvw)

One cross-sectional study was identified where population-based studies had been pooled to obtain data on almost 12,000 over 18-year-olds from the general population in the US who had been prospectively screened for heart valve disorders. [[44]](https://paperpile.com/c/9wtXWX/5bn3) Cross-sectional data from the NHS Abdominal Aortic Aneurysm Screening Programme (NAAASP), which invites all men in their 65th year for ultrasound screening, was used to estimate population incidence of AAA. [[42]](https://paperpile.com/c/9wtXWX/G0ue) The randomised controlled trial tested a community-based screening programme for AF in 2005.[38] This involved using prevalence of known AF from EHR screening as well as screening with pulse check and ECGs within a random sample of 14,802 over 65-year-olds living in the West Midlands, UK.

No studies were identified that had screened for all anxiety disorders, but the European Study of the Epidemiology of Mental Disorders used a diagnostic interview in general population surveys of approximately 150,000 adults in 26 countries to determine cross-sectional population prevalence of generalised anxiety disorder in 18–99-year-olds between 2001-2012. [[45]](https://paperpile.com/c/9wtXWX/GKmr) There were no screening studies for stroke or T1DM as patients with these conditions are usually affected significantly enough to present to hospital in the acute stages, and so screening is not conducted.

**Additional table 1: Prevalence of cardio-renal metabolic and mental health conditions in UK primary care electronic health records**

| **Long term condition** | **Number of cases** | **Population denominator** | **UK primary care EHR Prevalence (%) (95% CI)** | **Source data** | **Year of data collection** | **Population** | **Age profile** | **Study type** | **Method of identifying cases** |
| --- | --- | --- | --- | --- | --- | --- | --- | --- | --- |
| **Heart failure** | 533,186 | 59,596,704 | 0.89  (0.89-0.90) | QOF [[18]](https://paperpile.com/c/9wtXWX/0xP04) | 2019-2020 | England | All | Nationally reported data | Read/SNOMED codes |
| **Atrial fibrillation** | 1,221,913 | 59,596,704 | 2.05  (2.05-2.05) | QOF [[18]](https://paperpile.com/c/9wtXWX/0xP04) | 2019-2020 | England | All | Nationally reported data | Read/SNOMED codes |
| **Stroke and TIA** | 1,070,704 | 59,596,704 | 1.80  (1.79-1.80) | QOF [[18]](https://paperpile.com/c/9wtXWX/0xP04) | 2019-2021 | England | All | Nationally reported data | Read/SNOMED codes |
| **Hypertension** | 8,393,073 | 59,596,704 | 14.08 (14.07-14.09) | QOF [[18]](https://paperpile.com/c/9wtXWX/0xP04) | 2019-2020 | England | All | Nationally reported data | Read/SNOMED codes |
| **Ischaemic heart disease** | 1,842,008 | 59,596,704 | 3.09  (3.09-3.10) | QOF [[18]](https://paperpile.com/c/9wtXWX/0xP04) | 2019-2020 | England | All | Nationally reported data | Read/SNOMED codes |
| **Peripheral vascular disease** | 355,712 | 59,596,704 | 0.60  (0.59-0.60) | QOF [[[[18]](https://paperpile.com/c/9wtXWX/0xP04)](https://paperpile.com/c/9wtXWX/0xP04) | 2019-2021 | England | All | Nationally reported data | Read/SNOMED codes |
| **Heart valve disorders** | 8,416 | 516,007 | 1.63  (1.60-1.67) | THIN [[28]](https://paperpile.com/c/9wtXWX/5Ejz) | 2008 | UK | 65-95 years | Cohort study using EHRs | Read codes |
| **Aortic aneurysm** | No available data | | | | | | | | |
| **Type 1 diabetes** | 7,750 | 2,466,364 | 0.31 (0.31-0.32) | THIN [[69]](https://paperpile.com/c/9wtXWX/UkQV) | 2007 | UK | All | Cross-sectional study using EHRs with an algorithm applied to refine case identification | Read codes, coded records of medication for diabetes, and blood glucose measures. |
| **All diabetes** | 3,405,803 | 48,131,401 | 7.08 (7.07-7.08) | QOF [[18]](https://paperpile.com/c/9wtXWX/0xP04) | 2019-2021 | England | Over 17 years | Nationally reported data | Read/SNOMED codes |
| **Chronic kidney disease** | 1,923,862 | 47,503,067 | 4.05 (4.04-4.06) | QOF [[18]](https://paperpile.com/c/9wtXWX/0xP04) | 2019-2021 | England | Over 18 years | Nationally reported data | Read/SNOMED codes |
| **Depression** | 5,489,517 | 47,503,067 | 11.56 (11.55-11.57) | QOF [[18]](https://paperpile.com/c/9wtXWX/0xP04) | 2019-2022 | England | Over 18 years | Nationally reported data | Read/SNOMED codes |
| **Anxiety** | 108,495 | 1,516,481 | 7.15  (7.11-7.20) | THIN [[33]](https://paperpile.com/c/9wtXWX/3wqb) | 2002-2004 | UK | 10–79 years | Cross-sectional study using EHRs | Read codes |
| **Bipolar disorder** | 36,187 | 10,136,415 | 0.36  (0.35-0.36) | IMRD [[29]](https://paperpile.com/c/9wtXWX/fPDM) | 2018 | UK | Over 6 years | Cross-sectional study using EHRs | Read codes |
| **Eating disorder** | 6,348 | 1,135,038 | 0.56  (0.55-0.57) | CPRD GOLD [[30]](https://paperpile.com/c/9wtXWX/gqcW) | 2004-2014 | England | 11-24 years | Cross-sectional study using EHRs | Read/SNOMED codes |
| **Schizophrenia** | 59,150 | 16,900,000 | 0.35  (0.35-0.35) | CPRD GOLD [[31]](https://paperpile.com/c/9wtXWX/Wsac) | 2014 | UK | All | Cross-sectional study using EHRs | Read/SNOMED codes |
| **PTSD** | No relevant studies found | | | | | | | | |
| **Drug/alcohol misuse** | No relevant studies found | | | | | | | | |

PTSD - Post Traumatic Stress Disorder, TIA - Transient Ischaemic Attack, QOF - Quality and Outcomes Framework, CRPD - Clinical Practice Research Datalink, THIN - The Health Improvement Network, IMRD - IQVIA Medical Research Data , EHR - Electronic Healthcare Records, UK - United Kingdom

**Additional table 2: Self-reported doctor-diagnosed prevalence of cardio-renal-metabolic and mental health conditions in the UK population**

| **Long term condition** | **Number of cases** | **Population denominator** | **Doctor diagnosed prevalence (%) (95% CI)** | **Source data** | **Study type** | **Year of data collection** | **Population** | **Age profile** | **Data collection method** | **Method for identifying cases** |
| --- | --- | --- | --- | --- | --- | --- | --- | --- | --- | --- |
| **Heart failure (HF)** | 361 | 14,986 | 2.41  (2.17-2.67) | NHANES [36] | National household survey | 2017- 2020 | USA | Over 20 years | Self-reports in national survey representative of US population | "Has a doctor or other health professional ever told {you/SP} that {you/s/he} . . .had congestive heart failure?" |
| **Atrial fibrillation** | No relevant studies found | | | | | | | | | |
| **Stroke** | 189 | 7,997 | 2.36  (2.03-2.70) | HSE [[34]](https://paperpile.com/c/9wtXWX/3x85) | National household survey | 2017 | England | Over 16 years | Questionnaire administered by a nurse - self reports in a representative of the population living in  private households in England | “Were you told by a doctor that you had a stroke?” |
| **Hypertension** | 647 | 4,166 | 15.54  (14.43-16.63) | HSE [[23]](https://paperpile.com/c/9wtXWX/JRDl) | National household survey | 2019 | England | Over 16 years | Questionnaire administered by a nurse - self reports in a representative of the population living in  private households in England | “Do you now have, or have you ever had high blood pressure (sometimes called  hypertension)?” |
| **Ischaemic heart disease (IHD)** | 335 | 7,997 | 4.19  (3.75-4.63) | HSE [[34]](https://paperpile.com/c/9wtXWX/3x85) | National household survey | 2017 | England | Over 16 years | Questionnaire administered by a nurse - self reports in a representative of the population living in  private households in England | “Yes” to either  “Were you told by a doctor that you had a heart attack (including myocardial infarction or coronary thrombosis)?”  Or  “Were you told by a doctor that you had angina” |
| **Peripheral vascular disease (PVD)** | 5,117 | 394,408 | 1.30  (1.26-1.33) | UK Biobank [[39]](https://paperpile.com/c/9wtXWX/rI11) | Cross- sectional analysis of large cohort study | 2006- 2010 | UK | 45 to 69 years | Interview of participants plus analysis of hospital inpatient electronic healthcare records. | Individuals were defined as having peripheral vascular disease based if they had at least one of the self-reported illness codes, OPCS procedure codes, or ICD codes from hospital inpatient health records. |
| **Heart valve disorder** | No relevant studies found | | | | | | | | | |
| **Aortic aneurysm** | No relevant studies found | | | | | | | | | |
| **Type 1 diabetes** | 218,670 | 52,172,792 | 0.42  (0.42-0.42) | National Diabetes Audit [[35]](https://paperpile.com/c/9wtXWX/JbtBB) | Nationally reported data | 2019/20 | UK | Over 19 years | From primary care and secondary care diabetes service records. | Read codes and SNOMED codes in primary care EHRs and patients on diabetes clinic lists in hospitals. |
| **All diabetes** | 581 | 8,205 | 7.08  (6.53-7.64) | HSE [[23]](https://paperpile.com/c/9wtXWX/JRDl) | National household survey | 2019 | England | Over 16 years | Questionnaire administered by a nurse - self reports in a representative of the population living in  private households in England | “‘Were you told by a doctor that you had diabetes?” |
| **Chronic kidney disease** | 100 | 5,047 | 1.99  (1.60-2.37) | HSE [[38]](https://paperpile.com/c/9wtXWX/yHeQ) | National household survey | 2016 | England | Over 16 years | Questionnaire administered by a nurse - self reports in a representative of the population living in  private households in England | “Have you ever been told by a doctor that you had chronic kidney disease?” |
| **Depression** | 1,576 | 7,542 | 20.90  (19.98-21.81) | APMS [[14]](https://paperpile.com/c/9wtXWX/3CO0) | National household survey | 2014 | England | Over 16 years | Survey of a large, stratified probability sample of the general population, covering people living in private households. Initial screening survey followed by structured assessment carried out by clinically trained interviewers with a subset of participants. | Participants were asked whether they thought that they had ever had depression. If they said "yes" they were asked if this had ever been diagnosed by a doctor and whether or not it was present in the past year. |
| **Generalised anxiety disorder** | 303 | 5,485 | 5.52  (4.92-6.13) | HSE [[37]](https://paperpile.com/c/9wtXWX/jqn1) | National household survey | 2014 | England | Over 16 years | Questionnaire administered by a nurse - self reports in a representative of the population living in  private households in England. | Participants asked if they had ever experienced “generalised anxiety disorder”. If participants said “yes” they were then asked whether they had been told by a doctor, psychiatrist or other professional that they had it. |
| **Bipolar disorder** | 31 | 5,485 | 0.56 (0.37-0.76) | HSE [[37]](https://paperpile.com/c/9wtXWX/jqn1) | National household survey | 2014 | England | Over 16 years | Questionnaire administered by a nurse - self reports in a representative of the population living in  private households in England | Participants asked if they had ever experienced “bipolar disorder”. If participants said “yes” they were then asked whether they had been told by a doctor, psychiatrist or other professional that they had it. |
| **Eating disorder** | 68 | 5,485 | 1.23  (0.95-1.53) | HSE [[31]](https://paperpile.com/c/9wtXWX/taej) | National household survey | 2019 | England | Over 16 years | Questionnaire administered by a nurse - self reports in a representative of the population living in  private households in England | Participants asked if they had ever experienced an “eating disorder”. If “yes” they were then asked whether they had been told by a doctor, psychiatrist or other professional that they had it. |
| **Schizophrenia** | 27 | 5,485 | 0.50  (0.31-0.68) | HSE [[37]](https://paperpile.com/c/9wtXWX/jqn1) | National household survey | 2014 | England | Over 16 years | Questionnaire administered by a nurse - self reports in a representative of the population living in  private households in England | Participants were asked if they had ever experienced “psychosis or schizophrenia”. If “yes” they were then asked whether they had been told by a doctor, psychiatrist or other professional that they had it. |
| **PTSD** | 102 | 5,485 | 1.86  (1.50-2.22) | HSE [[37]](https://paperpile.com/c/9wtXWX/jqn1) | National household survey | 2014 | England | Over 16 years | Questionnaire administered by a nurse - self reports in a representative of the population living in  private households in England | Participants were asked if they had ever experienced “post traumatic stress”. If participants said “yes” they were then asked whether they had been told by a doctor, psychiatrist or other professional that they had it. |
| **Drug/alcohol misuse** | 68 | 5,485 | 1.24  (0.95-1.53) | HSE [[37]](https://paperpile.com/c/9wtXWX/jqn1) | National household survey | 2014 | England | Over 16 years | Questionnaire administered by a nurse - self reports in a representative of the population living in  private households in England | Participants were asked if they had ever experienced “alcohol or drug dependence”. If participants said “yes” they were then asked whether they had been told by a doctor, psychiatrist or other professional that they had it. |

NHANES = National Health and Nutrition Examination Survey, HSE = Health survey for England, APMS = Adult Psychiatric Morbidity Survey

**Additional table 3: Screen-detected prevalence of cardio-renal-metabolic and mental health conditions in the UK population**

| **Long term condition** | **Number of cases** | **Population denominator** | **Screen- detected Prevalence (%) (95% CI)** | **Source data** | **Study type** | **Year of data collection** | **Population** | **Age profile** | **Data collection method** | **Method for identifying cases** |
| --- | --- | --- | --- | --- | --- | --- | --- | --- | --- | --- |
| **Heart failure (HF)** | 39 | 706 | 5.52  (3.84-7.21) | Galasko et al. [43] | Community based cohort study | 2000-2001 | Harrow, England | Over 45 years | Participants randomly selected from general practices in Harrow, England | Echocardiogram on random sample of population. Left ventricular systolic dysfunction defined as ejection fraction < 50%. |
| **Atrial fibrillation (AF)** | 1,264 | 14,802 | 8.54  (8.09-8.99) | Hobbs et al.[[41]](https://paperpile.com/c/9wtXWX/Kg1D) | Trial of a community screening programme | 2000-2005 | Birmingham, England | Over 65 years | Participants invited from a random sample of general practices in Birmingham, England | Search within EHRs for diagnosis of AF at baseline. Participants not known to have AF were systematically screened using a pulse check and ECG analysed by two cardiologists. |
| **Stroke** | No studies available, those with symptomatic strokes typically seek healthcare assessment. | | | | | | | | | |
| **Hypertension** | 1161 | 4,166 | 27.87  (26.51- 29.23) | HSE [[23]](https://paperpile.com/c/9wtXWX/JRDl) | National household survey | 2019 | England | Over 16 years | Questionnaire administered by a nurse in a representative sample of the population living in  private households in England. | Hypertension was defined as systolic blood pressure (SBP) of 140mmHg or higher, diastolic blood pressure (DBP) of 90mmHg or higher. Mean of second and third readings taken  1 min apart after 5 min rest and/or taking medicine to reduce blood pressure. |
| **Ischaemic Heart Disease (IHD)** | Public Health England (PHE) modelled prevalence estimates for England calculated at 7.40% (no frequency data available). | | | Eastern Region Public Health Observatory (ERPHO) [40] | Model based on Whitehall II, ELSA and other cohort studies | 2015 | England | 55-79 years | Modelling based on longitudinal data | Self-reported symptoms of angina and diagnosis of IHD |
| **Peripheral vascular disease (PVD)** | 78 | 7,845 | 0.99 (0.77-1.21) | HSE [[34]](https://paperpile.com/c/9wtXWX/3x85) | National household survey | 2017 | England | Over 16 years | Questionnaire administered by a nurse in a representative sample of the population living in  private households in England. | Self-reported symptoms suggestive of peripheral artery disease: pain in the calves on walking uphill that ceases within 10 minutes of stopping walking but no pain on sitting or standing. |
| **Heart valve disorders** | 298* adj. For age and sex | 11,911 | 2·5 (2·2–2·7) | Nkomo et al. [[44]](https://paperpile.com/c/9wtXWX/5bn3) | Pooled population- based studies | 2000 | USA | Over 18 years | Randomly sampled adults from general population | Echocardiogram - valve diseases diagnosed by cardiologist. |
| **Abdominal aortic aneurysm** | 2,318 | 238,107 | 0.97  (0.93-1.01) | NAAASP [[42]](https://paperpile.com/c/9wtXWX/GkJMu) | National screening programme | 2019 | UK | Men aged over 65 years | National screening programme, men 66 years and over invited for testing | Abdominal ultrasound scan finding of aorta measuring ≥3cm |
| **Type 1 Diabetes** | No studies available. Once symptomatic, patients are likely to be admitted to hospital. | | | | | | | | | |
| **All diabetes** | 362 | 3,496 | 10.37 (9.24-11.36) | HSE [[23]](https://paperpile.com/c/9wtXWX/JRDl) | National household survey | 2019 | England | Over 16 years | Questionnaire and blood test taken by a nurse in a representative sample of the population living in  private households in England. | Participants who reported having a diagnosis of diabetes and/or with a glycated haemoglobin (HbA1c) measurement of 48 mmol/mol or above. |
| **Chronic kidney disease (CKD)** | 176 | 3,464 | 5.08 (4.35-5.81) | HSE [[38]](https://paperpile.com/c/9wtXWX/yHeQ) | National household survey | 2016 | England | Over 16 | Questionnaire and blood test taken by a nurse in a representative sample of the population living in  private households in England. | Kidney function tests were measured in 2003 and 2016 (serum creatinine, cystatin C and urinary albumin/creatinine ratio (ACR)). CKD was defined as eGFR <60 and/or albuminuria. |
| **Depression** | 2096.676 | 7,542 | 27.8  (26.79- 28.81) | APMS [[14]](https://paperpile.com/c/9wtXWX/3CO0) | National household survey | 2014 | England | Over 16 | Questionnaire administered by a clinically trained interviewer in a representative of the population living in  private households in England. | Participants were given a description of depression and asked whether they thought that they had ever had this. |
| **Anxiety** | 4,417 | 81,836 | 5.40 (5.24-5.55) | European Study of the Epidemiology of Mental Disorders [[45]](https://paperpile.com/c/9wtXWX/GKmr) | Cross- sectional international survey | 2000 | High income countries | 18-99 years | Adults from representative households were interviewed face-to-face in the community. | WHO Composite International Diagnostic Interview (CIDI), diagnosed with generalised anxiety disorder based on DSM-5 criteria. |
| **Bipolar disorder** | 139 | 7,076 | 1.96 (1.64-2.29) | APMS [[14]](https://paperpile.com/c/9wtXWX/3CO0) | National household survey | 2014 | England | Over 16 years | Survey of a large, stratified probability sample of the general population, covering people living in private households. Initial screening survey followed by structured assessment carried out by clinically trained interviewers with a subset of participants. | Mood Disorder Questionnaire (Hirschfeld et al. 2000) Cases were identified if participants displayed 7+ symptoms of bipolar disorder at the same time and this caused problems in their life. |
| **Eating disorder** | 312 | 7,398 | 4.22 (3.77-4.70) | HSE [[31]](https://paperpile.com/c/9wtXWX/JRDl) | National household survey | 2019 | England | Over 16 years | Questionnaire administered by a nurse in a representative sample of the population living in private households in England. | Score of 2 or more on the SCOFF scale (designed to screen for *possible* eating disorders) AND reported that their feelings about food had a significant negative impact on their lives. |
| **Schizophrenia** | 108 | 14,949 | 0.72 (0.59-0.86) | APMS [[14]](https://paperpile.com/c/9wtXWX/3CO0) | National household survey | 2014 | England | Over 16 years | Survey of a large, stratified probability sample of the general population, covering people living in private households. Initial screening survey followed by structured assessment carried out by clinically trained interviewers with a subset of participants. | Questionnaire identified anyone currently taking any antipsychotic medication, reporting an inpatient stay for a mental or emotional problem in the past three months, or having been admitted to a hospital or ward specialising in mental health problems at any time. Additionally, anyone reporting auditory hallucinations, symptoms suggestive of psychotic disorder (such as mood swings) and/or discussing such symptoms with a GP in the past year, or if they thought they ever had any of a list of psychiatric disorders.  Diagnoses were confirmed or refuted in using the SCAN interview (WHO 1999) - for psychotic disorders. |
| **Post-traumatic stress disorder (PTSD)** | 311 | 7,066 | 4.40  (3.92-4.88) | APMS [[14]](https://paperpile.com/c/9wtXWX/3CO0) | National household survey | 2014 | England | Over 16 | Survey of a large, stratified probability sample of the general population, covering people living in private households. Initial screening survey followed by structured assessment carried out by clinically trained interviewers with a subset of participants. | 7-item PTSD Checklist – Civilian (PCL-C) in the  self-completion part of the interview. Those with a score of 50 or more and meeting Diagnostic Statistical Manual (DSM) criteria for PTSD were identified as screening positive for PTSD. |
| **Alcohol misuse** | 223 | 7,218 | 3.09 (2.69-3.49) | APMS [[14]](https://paperpile.com/c/9wtXWX/3CO0) | National household survey | 2014 | England | Over 16 | Survey of a large, stratified probability sample of the general population, covering people living in private households. Initial screening survey followed by structured assessment carried out by clinically trained interviewers with a subset of participants. | AUDIT questionnaire scores >16 used to indicate harmful drinking and at least mild dependence. |
| **Drug dependence** | 213 | *6,925* | 3.07 (2.67-3.48) | APMS [[14]](https://paperpile.com/c/9wtXWX/3CO0) | National household survey | 2014 | England | Over 16 | Survey of a large, stratified probability sample of the general population, covering people living in private households. Initial screening survey followed by structured assessment carried out by clinically trained interviewers with a subset of participants. | For each of eight drug types (cannabis, amphetamines, crack, cocaine, ecstasy, tranquillisers, opiates and volatile substances), reported use in the past year  was followed by five questions based on the Diagnostic Interview Schedule and designed to assess symptoms of drug dependence. These  questions asked about the past month and year, and covered:  • Daily use for 2 weeks or more  • Sense of need or dependence  • Inability to abstain  • Increased tolerance, and  • Withdrawal symptoms.  A positive response to any of the items was used as an indicator of possible drug dependence. |

HSE = Health survey for England, APMS = Adult Psychiatric Morbidity Survey, NAAASP = NHS Abdominal Aortic Aneurysm Screening Programme,

PHE = Public Health England

**Further references**

69. de Lusignan S, Liaw S-T, Dedman D, Khunti K, Sadek K, Jones S. An algorithm to improve diagnostic accuracy in diabetes in computerised problem orientated medical records (POMR) compared with an established algorithm developed in episode orientated records (EOMR). J Innov Health Inform. 2015;22: 255–264.
